# Impaired dynamic interaction of axonal endoplasmic reticulum and ribosomes contributes to defective stimulus–response in spinal muscular atrophy

**DOI:** 10.1186/s40035-022-00304-2

**Published:** 2022-06-02

**Authors:** Chunchu Deng, Sebastian Reinhard, Luisa Hennlein, Janna Eilts, Stefan Sachs, Sören Doose, Sibylle Jablonka, Markus Sauer, Mehri Moradi, Michael Sendtner

**Affiliations:** 1 Institute of Clinical Neurobiology, University Hospital Wuerzburg, 97078 Würzburg, Germany; 2 Department of Biotechnology and Biophysics, Biocenter, Julius-Maximilians-University Wuerzburg, 97074 Würzburg, Germany

**Keywords:** Spinal muscular atrophy, Presynaptic ER dynamics, Dynamics of ribosomal assembly, BDNF stimulation

## Abstract

**Background:**

Axonal degeneration and defects in neuromuscular neurotransmission represent a pathological hallmark in spinal muscular atrophy (SMA) and other forms of motoneuron disease. These pathological changes do not only base on altered axonal and presynaptic architecture, but also on alterations in dynamic movements of organelles and subcellular structures that are not necessarily reflected by static histopathological changes. The dynamic interplay between the axonal endoplasmic reticulum (ER) and ribosomes is essential for stimulus-induced local translation in motor axons and presynaptic terminals. However, it remains enigmatic whether the ER and ribosome crosstalk is impaired in the presynaptic compartment of motoneurons with Smn (survival of motor neuron) deficiency that could contribute to axonopathy and presynaptic dysfunction in SMA.

**Methods:**

Using super-resolution microscopy, proximity ligation assay (PLA) and live imaging of cultured motoneurons from a mouse model of SMA, we investigated the dynamics of the axonal ER and ribosome distribution and activation.

**Results:**

We observed that the dynamic remodeling of ER was impaired in axon terminals of Smn-deficient motoneurons. In addition, in axon terminals of Smn-deficient motoneurons, ribosomes failed to respond to the brain-derived neurotrophic factor stimulation, and did not undergo rapid association with the axonal ER in response to extracellular stimuli.

**Conclusions:**

These findings implicate impaired dynamic interplay between the ribosomes and ER in axon terminals of motoneurons as a contributor to the pathophysiology of SMA and possibly also other motoneuron diseases.

**Supplementary Information:**

The online version contains supplementary material available at 10.1186/s40035-022-00304-2.

## Background

Spinal muscular atrophy (SMA) is an autosomal recessive neuromuscular disease, which is caused by mutations in the *SMN1* gene. SMA is characterized by degeneration of spinal motoneurons and muscle atrophy, ultimately leading to lethality [1]. Spinal motoneurons are highly polarized cell types, with a long axon and multiple dendrites, posing a great challenge for the regulation of protein homeostasis at synapses. The survival motor neuron (SMN) protein plays a role in the assembly of small nuclear ribonucleoproteins (snRNPs) implicated in pre-mRNA splicing [2, 3]. Moreover, interaction of SMN with RNA-binding proteins in neuronal axons has provided strong evidence of its central role in assembly and function of ribonucleoprotein particles involved in axonal mRNA transport [4–6] and local translation [7, 8]. This is reflected by alterations in the composition of the axonal transcriptome in Smn-deficient motoneurons [5]. mRNA localization and intra-axonal protein synthesis provide conserved mechanisms for a rapid modulation of the local proteome in response to extracellular cues, thereby regulating diverse cellular functions such as synaptogenesis, plasticity, and axon regeneration [9–11]. In axonal growth cones, ribosomes have been shown to react rapidly to external signals by closely interacting with activated receptors, leading to translation of receptor-specific mRNAs in growing retinal ganglion cell axons [12]. Interestingly, in motoneuron-like NSC34 cells as well as in brain and spinal cord tissues, SMN has been found associated with a subpopulation of ribosomes and this SMN/ribosome interaction appears essential for the translation of SMA-related transcripts [8]. In neuronal axons, ER forms a highly dynamic network that constantly contacts the plasma membrane and interacts with other organelles [13–16]. These interactions appear highly important for organizing axon terminals in developing neurons. A coordinated interplay between ER and organelles such as mitochondria [17, 18] and ribosomes [19, 20] is required to maintain cellular functions and plasticity [21]. The interaction between axonal ER and ribosomes seems to be essential for the neosynthesis and integration of transmembrane proteins including ion channels and cell surface receptors, leading to an enhanced cellular response and plasticity [22–25]. Mutations that impair ER shaping and function or alter the ER/organelles interaction can cause neurodegenerative diseases such as hereditary spastic paraplegia (HSP), Alzheimer’s disease (AD), Charcot–Marie–Tooth disease (CMT), Parkinson’s disease and amyotrophic lateral sclerosis (ALS) [26]. Thus, whether loss of SMN could associate with impaired ER dynamics and possibly also the assembly of ribosomes in the axon, thereby affecting the cellular response to extracellular stimulation, remains elusive.

In this study, we investigated the dynamic remodeling of axonal ER as well as the interaction of ribosomes with ER in axon terminals of Smn-deficient motoneurons in response to the brain-derived neurotrophic factor (BDNF)/ tropomyosin receptor kinase B (TrkB) signaling.

## Materials and methods

### Animals

The type I SMA mouse model was generated by mating a transgenic mouse expressing two copies of the human *SMN*2 gene onto a mouse null *Smn*−/− background [27]. The SMA mice were housed in the animal facility of the Institute of Clinical Neurobiology at the University Hospital of Wuerzburg. All experiments were performed in agreement with the regulations on animal protection of the German federal law and of the Association for Assessment and Accreditation of Laboratory Animal care.

### Primary motoneuron culture

Isolation and culture of primary embryonic mouse motoneurons were carried out as previously described [28]. Lumbar spinal cords were dissected from E13.5 mouse embryos. After removal of meninges and dorsal root ganglia, the ventrolateral parts of the collected spinal cords were digested with trypsin (CellSystems GmbH, Troisdorf, Germany). Cells were dispersed by trituration and motoneurons were enriched with anti-p75 antibody (MLR2, Abcam, Berlin, Germany) [28]. The motoneurons were plated on glass coverslips (A. Hartenstein GmbH, Wuerzburg, Germany) coated with poly-ornithine (PORN) (Merck-Millipore, Darmstadt, Germany) and laminin221/211 (CC085, Merck-Millipore, Darmstadt, Germany). Cells were maintained in Neurobasal medium (NB) (Gibco, Fisher Scientific GmbH, Schwerte, Germany) supplemented with 500 µM Glutamax, 2% B27 (ThermoFisher Scientific, Darmstadt, Germany), 2% heat-inactivated horse serum (Gibco, Fisher Scientific GmbH, Schwerte, Germany) and 5 ng/ml BDNF at 37 °C in a humidified CO_2_ incubator for 6 days. Medium was changed every second day.

### Plasmid constructs and lentiviral transduction

Lentiviral construct of pSIH-mCherry-KDEL, GFP-actin-IRES-mCherry-KDEL, or pSIH-GFP (used as control) was cloned into a pSIH vector backbone with CMV promotor as described previously [25]. GFP-SMN lentiviral construct was a gift from Changhe Ji (Institute of Clinical Neurobiology, University Hospital of Wuerzburg). Lentiviral particles were produced in HEK^293T^ cells (System Biosciences, 293TN Producer Cell Line) using pCMV-VSVG and pCMVΔR8.91 helper plasmids, as previously described [29]. For lentivirus transduction, motoneurons were incubated with lentiviral particles for 10 min at room temperature (RT) and plated afterwards.

### BDNF stimulation and puromycin incorporation assay

For BDNF pulse experiments, motoneurons were grown on coverslips and cultured with 5 ng/ml BDNF for 5 days in vitro. At day in vitro (DIV) 5, the BDNF-containing medium was removed, cells were washed twice with NB medium and cultured with NB medium supplemented with 2% horse serum and 2% B27 without BDNF overnight. At DIV6, the coverslips were first transferred onto a 37 °C hotplate, and 40 ng/ml BDNF was added directly into the culture medium and incubated for 10 s, 1-min or 10 min. In the BDNF-null condition, cells were incubated with the NB medium without BDNF for 10 s, 1-min or 10 min. When used at low concentrations, puromycin incorporation into nascent polypeptide chains enables direct visualization of the translation and thus can be applied to measure and quantify the amount of newly synthesized proteins in vitro [25, 30, 31]. Motoneurons were first deprived from BDNF overnight as described above. Then 10 µg/ml puromycin was added directly into the culture medium and incubated at 37 °C in a humidified CO_2_ incubator for 10 min, followed by BDNF stimulation in the presence of puromycin. After BDNF pulse application, cells were immediately fixed with 4% paraformaldehyde (PFA) and further processed for immunofluorescence labeling for puromycin.

### Surface TrkB staining assay

For visualization of TrkB presented exclusively at the cell surface, a live cell staining protocol was used. Cells were placed on ice and all the following staining steps were performed on ice. Antibody against TrkB (1:200, polyclonal rabbit anti-TrkB, Merck-Millipore, Darmstadt, Germany, 07-225) was diluted in cold NB medium supplemented with 2% B27 and 2% horse serum, added onto cells and incubated for 45 min. Following three washing steps with cold PBS, the cells were incubated with secondary antibody (1:300, Alexa Fluor 488 donkey anti-rabbit IgG (H + l) AffiniPure, BIOZOL, Eching, Germany, 711-545-152) diluted in cold NB medium supplemented with 2% B27 and 2% horse serum for 30 min. After 3 washes with cold PBS, cells were fixed with 4% PFA (Pierce^™^, ThermoFisher Scientific, Darmstadt, Germany) for 5 min on ice. Following TrkB surface staining, the cells were immunostained against synaptophysin 1 to visualize the growth cone boundaries. For synaptophysin 1 immunostaining, the permeabilization step was omitted and a standard staining protocol was applied as described below.

### Immunofluorescence assay

Motoneurons were fixed with 4% PFA for 10 min at RT. After 3 washes with PBS, the cells were permeabilized with 0.3% Triton X-100 in PBS for 10 min, washed with PBS and then incubated with block solution containing 10% donkey serum and 2% BSA for 1 h at RT. For Cav2.2 immunostaining, cells were fixed with 4% PFA for 5 min at RT, followed by 3 washes with PBS and permeabilized with 0.1% Triton X-100 in PBS for 5 min at RT. Cells were incubated with primary antibodies, which were diluted in the block solution at 4 °C overnight. On the next day, the cells were washed 3 times (each for 5 min) with TBST and incubated with secondary antibodies for 1 h at RT. For Ribosomal Protein L24 (RPL24) and Ribosomal Protein S6 (RPS6) co-staining, in some experiments 10 µg/ml cycloheximide was added to the NB medium containing BDNF or the NB medium without BDNF (for no BDNF condition) as well as to the 4% PFA buffer, to prevent ribosome disassembly. When compared to the experiments without cycloheximide, no differences in the number of RPL24/RPS6 co-clusters were observed after a 1-min BDNF stimulus. Nuclei were stained using DAPI. The following primary antibodies were used: polyclonal guinea pig antiserum RFP (Red fluorescent protein) (1:500, 390004, Synaptic Systems, Göttingen, Germany), monoclonal mouse anti-α-Tubulin (1:1000, T5168, Sigma-Aldrich, Darmstadt, Germany), monoclonal mouse anti-rRNA (Y10B) (1:500, MA116628, ThermoFisher Scientific, Darmstadt, Germany), polyclonal rabbit anti-eukaryotic elongation factor 2 (eEF2) (1:50, 23325, Cell Signaling Technology, Frankfurt, Germany), polyclonal rabbit anti-RPL24 (1:500, PA530157, ThermoFisher Scientific, Darmstadt, Germany), monoclonal mouse anti-S6 ribosomal protein (1:50, MA515123, ThermoFisher Scientific, Darmstadt, Germany), polyclonal guinea pig anti- N-type alpha-1B Ca^2+^ channel (1:250, 152305, Synaptic Systems, Göttingen, Germany), polyclonal rabbit anti-Tau (1:1000, T6402, Sigma-Aldrich, Darmstadt, Germany), monoclonal mouse anti-SMN (1:250, 610646, BD Biosciences, Heidelberg, Germany), polyclonal guinea pig anti-synaptophysin 1 antibody (1:1000, 101004, Synaptic Systems, Göttingen, Germany), polyclonal goat anti-TrkB (1:500, AF1494, Bio-Techne, Wiesbaden, Germany), monoclonal rabbit anti-pTrkA/B (1:500, 4619, Cell signaling, Frankfurt, Germany) and monoclonal mouse anti-puromycin (1:1000, MABE343, clone 12D10, Merck Millipore, Darmstadt, Germany). Secondary antibodies used were: Alexa Fluor 488 donkey anti-rabbit IgG (H + l) AffiniPure (711-545-152, BIOZOL, Eching, Germany), Alexa Fluor 488 donkey anti-mouse IgG (H + L) (A21202, Life Technologies, Darmstadt, Germany), Cy3 donkey anti-goat IgG (H + L) AffiniPure (705-165-147, BIOZOL, Eching, Germany), Cy3 donkey anti-guinea pig IgG (H + L) AffiniPure (706-165-148, BIOZOL, Eching, Germany), Cy3 goat anti-mouse IgG (H + L) AffiniPure (115-165-146, BIOZOL, Eching, Germany), Cy3 donkey anti-rabbit IgG (H + L) AffiniPure (711-165-152, BIOZOL, Eching, Germany), Cy5 donkey anti-rabbit IgG (H + L) AffiniPure (711-175-152, BIOZOL, Eching, Germany), Alexa Fluor 647 donkey anti-goat IgG (H + L) AffiniPure (705-605-003, BIOZOL, Eching, Germany), and Alexa Fluor 647 donkey anti-mouse IgG (H + L) highly cross-adsorbed (A31571, Invitrogen, Darmstadt, Germany). Alexa Fluor 647-conjugated Phalloidin (A22287, Invitrogen, Darmstadt, Germany) was used for F-actin labelling. All the secondary antibodies were diluted at 1:500 in TBST. For embedding, Aqua Poly/Mount (18606-20, Polysciences, Hirschberg, Germany) was used.

### Image acquisition with confocal and structured illumination microscopy (SIM)

For image acquisition, an Olympus Fluoview 1000 confocal system and an ELYRA S.1 SIM Zeiss from AG were used. The Olympus Fluoview 1000 was equipped with a 60× NA 1.35 oil objective and the ELYRA S.1 SIM was equipped with a Plan-Apochromat 63× NA 1.4 oil objective and 405 nm diode (50 mW), 488 nm OPSL (100 mW), 561 nm OPSL (100 mW) and 642 nm diode (150 mW) excitation lasers. The x–y–z resolution of the SIM microscope was ~ 120 nm. For each growth cone, the maximum projection images were created from five 0.12-µm z-stacks, which are shown for representative images. Quantification of colocalization was performed from single optical sections with a z-resolution of ~ 120 nm. Acquired raw 16-bit images from two or three channels were processed with a commercial software package (Zeiss) to reconstruct high-resolution images and channel alignment was performed on the reconstructed images using Fidulial markers (TetraSpeck^™^ Microspheres, 0.2 µm, fluorescent blue/green/orange/dark red, T7280, ThermoFisher Scientific, Darmstadt, Germany). Images were further processed for quantification using ImageJ-win64 (Bethesda, MD).

### Live cell imaging

For live cell imaging, motoneurons were seeded on µ-dishes 35 mm, high (81156, Ibidi), coated with PORN/laminin221/211, and cultured for 6 days. The motoneurons were transduced with lentiviruses expressing mCherry-ER to visualize dynamic movements of ER or with GFP-actin-IRES-mCherry-KDEL for rescue experiments. SiR-Actin (CY-SC006, Cytoskeleton, Frankfurt, Germany) was used to visualize actin movements in growth cone filopodia. For SiR-Actin staining, mCherry-ER-expressing neurons were incubated with 25 nmol of SiR-Actin for 10 min at 37 °C prior to live cell imaging. Cells were first washed 2× with pre-warmed Tyrode’s Solution (125 mM NaCl, 2 mM KCl, 2 mM CaCl_2_, 2 mM MgCl_2_, 30 mM glucose, 25 mM HEPES, pH 7.4). Next, 2 ml of Tyrode’s Solution and 5 ng/ml BDNF were added to the cells, and the µ-dishes were then placed into a Tokai Hit chamber for live cell imaging. Imaging was performed at 37 °C using an inverted TE2000 Nikon epifluorescence microscope equipped with a 60× 1.4 NA objective, heated stage chamber (TOKAI HIT CO, LTD), 5% CO_2_, perfect focus system, an Orca Flash 4.0 V2 camera (Hamamatsu Photonics) and Nikon Element image software. Then 12-bit images of 1.024 × 1.024 pixels were acquired at a speed of 2 s/frame for a total period of 15 min. For treatment experiments, 1 µg/ml cytochalasin D (CytoD) (Sigma-Aldrich; C2618) or 0.5 mg/ml DMSO (in control conditions) was added to the NB medium and incubated for 30 min at 37 °C in a cell culture incubator. Next, the NB medium was removed, cells were washed 2× with Tyrode’s Solution and imaged in 2-ml Tyrode’s Solution containing 5 ng/ml BDNF as well as 1 µg/ml CytoD or 0.5 mg/ml DMSO for live cell imaging.

### Proximity ligation assay (PLA)

Duolink^®^proximity ligation assay (PLA^®^) was used for detection of protein interaction between RPL24 (60S subunits) and RPS6 (40S subunits) after BDNF pulse application [32, 33]. Furthermore, RiboPuromycylation-PLA assay was carried out to detect actively translating ribosomes in growth cones in situ. A PLA signal is detected only when interacting proteins are in close proximity of < 40 nm [34]. Motoneurons were transduced with a GFP-expressing lentivirus to label the cell boundaries and cultured for 6 days in vitro. Cells were stimulated with BDNF for 1-min as described above, followed by a 10-min fixation at RT using 4% PFA. To prevent ribosomal disassembly, 10 µg/ml cycloheximide was included into BDNF or for the control group into the BDNF-free medium during the BDNF pulse, as well as into the PFA fixation buffer. After PBS wash, permeabilization was performed using 0.2% Triton X-100 for 10 min at RT. The PLA assay was conducted using Duolink^®^ In Situ Detection Reagents Orange (DUO92007, Sigma-Aldrich) following the manufacturer’s instruction. Briefly, cells were first transferred into a dark/humid chamber and incubated with Duolink blocking solution for 1 h at 37 °C. The primary antibodies were diluted in the Duolink antibody diluent and incubated with cells for 2 h at RT. The cells were washed first several times and then for 2 × 5 min with 1× Wash Buffer A (DUO82049, Sigma-Aldrich) at RT. PLA probes, anti-mouse MINUS (DUO92004) and anti-rabbit PLUS (DUO92002), were diluted 1:50 in Duolink antibody diluent and incubated with the cells for 1 h at 37 °C. After multiple washing steps with 1x Wash Buffer A, ligase (diluted 1:40 in ligation buffer) was added to the cells and incubated for 30 min at 37 °C. The cells were washed again several times with 1× Wash Buffer A and incubated with polymerase (diluted 1:80 in amplification buffer) for 100 min at 37 °C. The amplification step was stopped by 2 × 10 min washes at RT with 1× Wash Buffer B (DUO82049, Sigma-Aldrich). Finally, the cells were washed for 1-min with 0.01× Wash Buffer B and mounted using Duolink mounting medium (DUO82040, Sigma-Aldrich) and sealed using nail polish. All the incubation steps at 37 °C were carried out in a dark/humid chamber using a dry incubator (Binder BD 23). Samples were imaged using a confocal microscope or stored at − 20 °C. Cells in the control group were pretreated with 200 µg/ml puromycin for 10 min at 37 °C prior to the PLA assay. When used at high concentrations, puromycin releases the 80S subunits into 60S and 40S, thereby resulting in reduced detection of PLA signal [35]. Moreover, for in situ visualization of actively translating ribosomes, RiboPuromycylation assay (RPM) [36, 37] combined with PLA was used as described earlier [31]. Briefly, cells were first treated with 10 µg/ml puromycin for 5 min at 37 °C. Following 1-min BDNF stimulation in the presence of 10 µg/ml cycloheximide, the puromycylation reaction was stopped by a PBS/MgCl_2_/CaCl_2_ wash (1 x PBS pH 7.4, 1 mM MgCl_2_, 0.1 mM CaCl_2_) and cells were fixed for 10 min with 4% PFA in 4% sucrose in PBS-MC buffer containing 10 µg/ml cycloheximide at RT. For the BDNF-negative condition, cells received only NB medium containing 10 µg/ml cycloheximide for 1-min and proceeded as described for BDNF pulse condition. In the RPM assay, the addition of the translation elongation inhibitors, cycloheximide, prevents the dissociation of puromycylated peptides from the translating ribosomes. Cells were then permeabilized with 0.2% Triton X-100 for 10 min at RT and incubated with rabbit anti-RPL24 (1:500) and mouse anti-puromycin (1:1000) primary antibodies. The PLA assay was performed as described above.

### Two-color *d*STORM

For two-color *d*STORM experiments, motoneurons were first stimulated with BDNF for 1-min and fixed with 4% PFA for 10 min at RT. To prevent ribosome disassembly, 10 µg/ml of cycloheximide was added either together with BDNF, or alone in the case of the BDNF-negative condition, into the medium for the time of BDNF pulse, as well as into the 4% PFA buffer. After fixation, the cells were washed with PBS and permeabilized with 0.1% Triton X-100 for 10 min at RT and blocked with 5% BSA in PBS for 1 h at RT. The cells were incubated with primary antibodies to RPL24 (1:500) and RPS6 (1:50), or with antibodies to Y10B (1:500) and eEF2 (1:200) for 2 h at RT. After washing with PBS, secondary antibodies including Alexa Fluor 532 goat anti-rabbit IgG (H + L) cross-adsorbed (1:200, ThermoFisher, A11009), CF568 donkey anti-rabbit IgG (H + L) highly cross-adsorbed (1:200, Merck, SAB4600076) and Alexa Fluor 647 donkey anti-mouse IgG (H + L) highly cross-adsorbed (1:800, Invitrogen, A31571) were added and incubated for 1 h at RT. An extra post-fixation step with 4% PFA (10 min at RT) was performed to increase the signal-to-noise ratio for two-color *d*STORM imaging. The samples were kept in PBS at 4 °C before imaging. Sample imaging was performed in the presence of a reducing agent to enable reversible photoswitching of the fluorophores. The imaging buffer consisted of 100 mM β-mercaptoethylamine (Sigma-Aldrich) in PBS (pH 7.4), as previously described [38]. Two-color *d*STORM measurements were performed on an inverted fluorescence wide-field microscope (Olympus IX-71, Hamburg, Germany) with an oil-immersion objective (APON 60×; numerical aperture 1.49; Olympus) and a nose-piece stage (IX2-NPS, Olympus) to reduce vibrations and drift during imaging. For excitation of Alexa Fluor 647 and Alexa Fluor 532, a 639-nm laser, a 514-nm laser (both OPSL, Genesis MX STM Series, Coherent, Dieburg, Germany) and a dichroic mirror (ZT405/514/635rpc, Chroma) were used. Another dichroic mirror (630 DCXR customized, Chroma, Augsburg, Germany) and two different bandpass filters (582/75 and 679/41 BrightLine series, Semrock, Tübingen, Germany) were placed in the detection path in front of two separate electron-multiplying charge-coupled device (EMCCD) cameras (iXon Ultra 897; Andor, Frankfurt, Germany) which detected the emission light. For two-color images with CF568, a 558-nm laser, a dichroic mirror (FF410/504/582/669, Chroma) and a 607/70 bandpass filter (Brightline, Semrock) were used. The two channels were imaged sequentially with an exposure time of 20 ms, 15,000 frames, Total Internal Reflection Fluorescence illumination and an irradiation intensity of 3–5 kW/cm^2^. These single molecule localization data were reconstructed with a pixel size of 10 nm using the open-source software rapidSTORM 3.3. Chromatic aberration was corrected by imaging 0.1-µm fluorescent microspheres (T7279, ThermoFisher Scientific, Darmstadt, Germany) after each measurement and creating an alignment matrix with the ImageJ plugin bUnwarpJ [39]. The matrix was then applied to the reconstructed two-color images.

### Western blotting

Motoneurons were isolated from *Smn*+*/*+*;SMN2tgtg* and *Smn*−/−*;SMN2tgtg* mice and cultured on PORN/laminin221/211-coated 24-well cell culture dishes. At DIV6, motoneurons were first deprived from BDNF overnight and then stimulated with 100 ng/ml BDNF for 10 min. Then the cells were lysed directly in 2× Laemmli buffer (125 mM Tris, pH 6.8, 10% SDS, 50% glycerol, 25% β-mercaptoethanol and 0.2% bromophenol blue), the lysates were boiled at 99 °C for 5 min, and proteins were separated on a 4%–12% gradient SDS-PAGE gel. Following protein transfer onto a PVDF membrane (BioRad, Feldkirchen, Germany) via the wet blot transfer system and membrane blocking with 5% milk, the membranes were probed with primary antibodies on a shaker at 4 °C overnight. After three washes with TBST, secondary antibodies were incubated for 1 h at RT. Following three 15-min washes with TBST, the membranes were incubated with secondary antibodies for 1 h at RT, washed three times with TBST, and developed using Amersham ECL^™^ Western blotting detection reagent (ThermoFisher Scientific, Darmstadt, Germany) and X-ray films (Fuji super RX). The primary antibodies used were monoclonal mouse anti-SMN (1:3000, BD Biosciences, 610646), polyclonal rabbit anti-Calnexin (1:3000, Enzo Life Sciences, ADI-SPA-860-F), polyclonal goat anti-TrkB (1:1000, Bio-Techne, AF 1494), monoclonal mouse anti-GAPDH (1:1000, Sigma-Aldrich, CB1001), monoclonal rabbit anti-pTrkA/B (1:1000, Cell signaling, 4619) and monoclonal mouse anti-β-Tubulin-III (1:4000, Sigma-Aldrich, T8578). The secondary antibodies used were peroxidase AffiniPure donkey anti-goat IgG (H + L) (1:10,000, 705-035-003, BIOZOL, Eching, Germany), peroxidase AffiniPure goat anti-rabbit IgG (H + L) (1:10,000, 111-035-144, BIOZOL, Eching, Germany) and peroxidase AffiniPure goat anti-mouse IgG (H + L) (1:10,000, 115-035-003, BIOZOL, Eching, Germany).

### Data analysis

For images captured with confocal microscopy, unprocessed raw images were used to quantify immunofluorescence signals by measuring the mean gray values after background subtraction using ImageJ-win64. The numbers of co-clusters of RPL24/RPS6 and RPL24/RPS6/mCherry-ER or Y10B/eEF2 and Y10B/eEF2/mCherry-ER shown in Figs. 3 and 4 were calculated by the JACoP colocalization plugin from ImageJ-win64. This colocalization analysis, which is broadly used for evaluating subcellular structures, relies on object-based approaches which takes into account the nature of the colocalization, the size, the form and the intensity distribution of fluorescent signals [40]. Linear contrast and brightness adjustment was applied to each channel using a defined threshold. Regions of interest (ROIs) within the growth cones were defined as the entire area labeled with individual markers RPL24, RPS6 or mCherry-ER. The number of RPL24, RPS6 and mCherry-ER co-clusters or Y10B, eEF2 and mCherry-ER co-clusters was computed as the number of colocalizing particles from two analyzed channels using JACoP colocalization analysis based on “Objects based methods” and “Centre of mass”. To quantify ER dynamics, Image Correlation Spectroscopy implemented in Python was applied as previously described [25, 41]. For PLA assay, a threshold was applied to all the images and the number of dots per growth cone was calculated using the “analyze particles” function from ImageJ-win64. Linear contrast enhancement was applied to all representative images using Adobe Photoshop CS4 (San Jose, CA) for a better visibility.

### Statistical analysis

All statistical analyses were performed with GraphPad Prism 8 software (San Diego, CA). One-way ANOVA with Dunn’s post-hoc test and Two-way ANOVA with Tukey multiple comparison post-hoc test were used to analyze statistical significance among multiple groups and Mann Whitney test for comparison between two groups. Data are shown in scatter dot plots with mean ± SEM, unless otherwise indicated.

## Results

### Dynamic movements of the axonal ER are impaired in Smn-deficient motoneurons

**Fig. 1:**
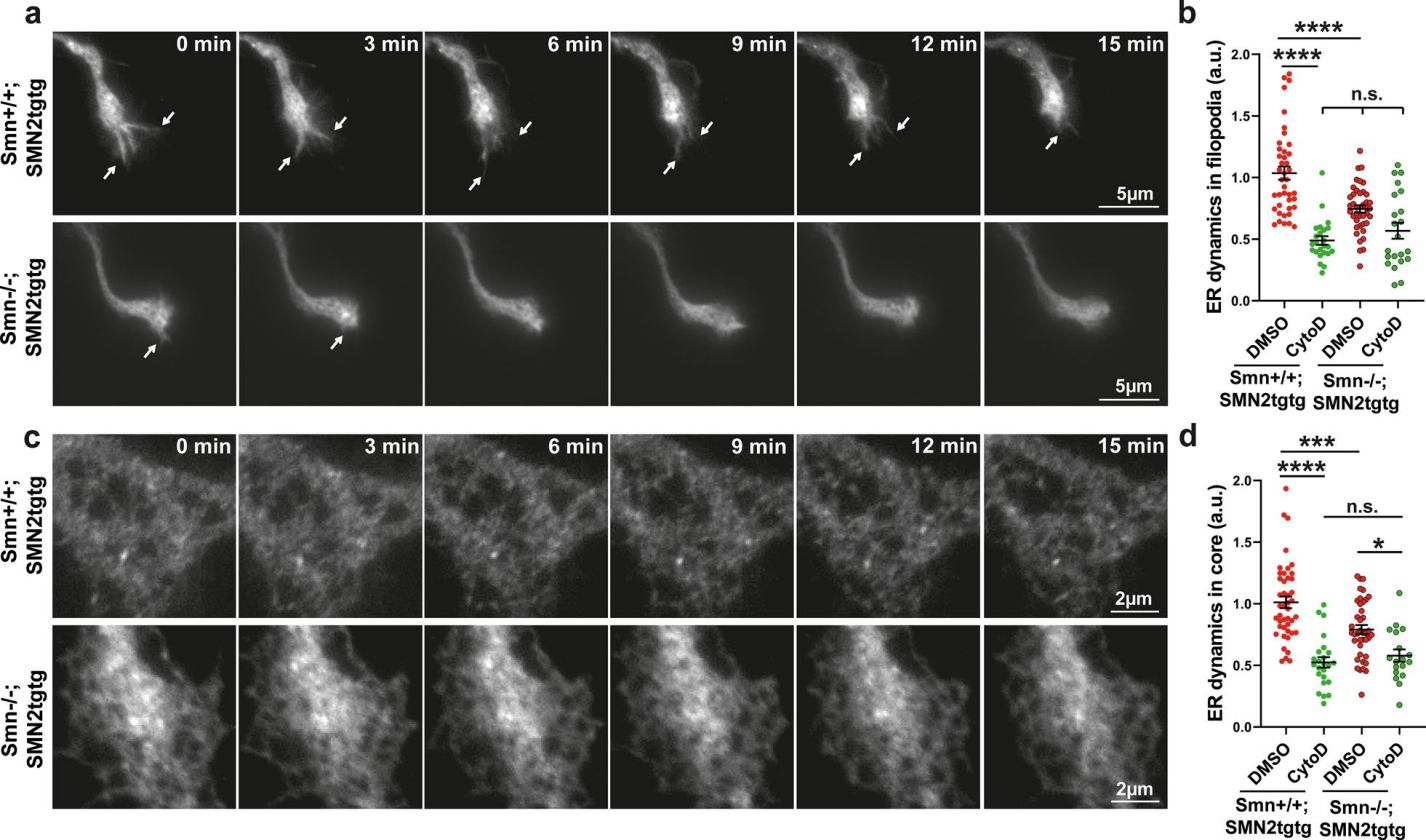
ER dynamic movements are disturbed in growth cones of Smn-deficient motoneurons. **a** Representative time-lapse images of growth cone filopodia from cultured *Smn*+*/*+*;SMN2tgtg* (control) and *Smn*−/−*;SMN2tgtg* motoneurons expressing mCherry-ER. Arrows indicate movements of ER in filopodia. **b** Quantitative Image Correlation Spectroscopy analysis of ER dynamics in growth cone filopodia shows reduced ER movements in *Smn*−/−*;SMN2tgtg* motoneurons, compared to control (****P < 0.0001; *n* = 41 cells from 6 independent experiments). CytoD-treatment results in reduced filopodia ER dynamics in *Smn*+*/*+*;SMN2tgtg* compared to DMSO-treated *Smn*+*/*+*;SMN2tgtg neurons* (****P < 0.0001). ER movements were also reduced in filopodia of CytoD-treated *Smn*−/−*;SMN2tgtg* compared to DMSO-treated *Smn*−/−*;SMN2tgtg* neurons (n.s., *P* = 0.0628; *n* = 22–41 cells from 3 independent experiments). **c** Representative time-lapse images of growth cone core from cultured *Smn*+*/*+*;SMN2tgtg* and *Smn*−/−*;SMN2tgtg* motoneurons expressing mCherry-ER. **d** Quantification of ER dynamics shows reduced ER movements in the core of *Smn*−/−*;SMN2tgtg* growth cones compared to control (***P = 0.0009; *n* = 40–44 cells from 6 independent experiments). ER movements are reduced in the growth cone core of CytoD-treated *Smn*+*/*+*;SMN2tgtg* compared to DMSO-treated *Smn*+*/*+*;SMN2tgtg* neurons (****P < 0.0001; *n* = 24–44 cells). CytoD treatment also reduces ER movements in the core of *Smn*−/−*;SMN2tgtg* growth cones (*P = 0.0235; *n* = 18–40 cells from 3 independent experiments)*.* All data are normalized to *Smn*+*/*+*;SMN2tgtg* control. Data are presented in scatter dot plot; error bars represent mean ± SEM. Statistical analyses were done by Two-way ANOVA with Tukey multiple comparison post-hoc test

**Fig. 2:**
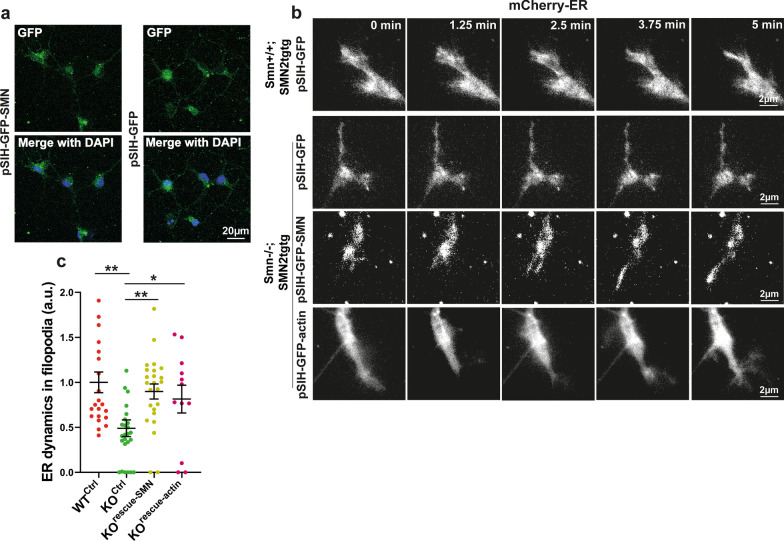
Overexpression of either SMN or actin rescues the impaired axonal ER dynamics in Smn-deficient motoneurons. **a** Motoneurons that were transduced with pSIH-GFP-SMN or pSIH-GFP lentiviruses were identified via GFP expression. **b** Representative time lapse images of ER movements in growth cone filopodia of *Smn*+*/*+*;SMN2tgtg* neurons co-expressing GFP and mCherry-ER (WT^Ctrl^), *Smn*−/−*;SMN2tgtg* neurons co-expressing GFP and mCherry-ER (KO^Ctrl^), *Smn*−/−*;SMN2tgtg* neurons co-expressing GFP-SMN and mCherry-ER (KO^rescue−SMN^) and *Smn*−/−*;SMN2tgtg* neurons co-expressing GFP-actin and mCherry-ER (KO^rescue−actin^). **c** ER dynamics are higher in growth cone filopodia of KO^rescue−SMN^ compared to the KO^Ctrl^ group (**P = 0.0016 for WT^Ctrl^ vs. KO^Ctrl^; **P = 0.0011 for KO^Ctrl^ vs. KO^rescue−SMN^; *n* = 22–26 cells from 3 independent experiments). ER dynamics are increased in filopodia of KO^rescue−actin^ motoneurons compared to the KO^Ctrl^ group (*P = 0.0468; *n* = 12–26 filopodia from 3 independent experiments). All data are normalized to WT^Ctrl^ control. Data are presented in scatter dot plot; error bars represent mean ± SEM. Statistical analyses were done by One-way ANOVA with Dunn’s post-hoc test

Several studies have shown that mutations in ER shaping proteins that affect tubular ER morphogenesis and dynamics cause neurological disorders including CMT, HSP and AD [26, 42, 43] and are responsible for axonopathies in ALS [44] and other neurodegenerative diseases [26]. In addition to ER shaping proteins, actin and microtubule cytoskeleton [45] as well as their associated proteins play an important role in ER dynamic movements in dendrites [46] and distal axons [25, 47]. Loss of function of the SMN protein correlates with reduced F-actin levels in axon terminals [6, 48] and could therefore be associated with impaired dynamic remodeling of the ER in this compartment. To investigate this, we transduced cultured *Smn*+*/*+*;SMN2tgtg* and *Smn*−/−*;SMN2tgtg* motoneurons with lentiviral particles expressing the ER marker mCherry-KDEL (mCherry-ER) [49] and performed live cell imaging of the ER in axonal growth cones (Fig. 1a–d). Quantification of ER movements in axonal growth cone filopodia revealed that the ER dynamic movements were significantly reduced in the growth cone filopodia of Smn-deficient motoneurons (Fig. 1a, b; Additional file 2: Video S1) compared to the *Smn*+*/*+*;SMN2tgtg* control (Fig. 1a, b; Additional file 3: Video S2). Similarly, in the growth cone core, ER movements were significantly decreased in *Smn*−/−*;SMN2tgtg* motoneurons (Fig. 1c, d; Additional file 4: Video S3; Additional file 5: Video S4). We recently showed that the dynamic remodeling of ER in axonal growth cones of motoneurons depends mainly on actin and its motor protein myosin VI [25]. Thus, we wondered whether such actin-dependent dynamics of the axonal ER are disturbed in SMA. To this aim, we performed pharmacological inhibition of F-actin polymerization using CytoD (30-min incubation with 1 µg/ml CytoD) and measured ER movements in the growth cone. In *Smn*+*/*+*;SMN2tgtg* growth cones, CytoD treatment severely reduced ER movements in both filopodia and core (Fig. 1b, d). In *Smn*−/−*;SMN2tgtg* growth cones, CytoD treatment also resulted in significant decreases in core ER movements and a slight but not significant decrease in filopodia ER movements (Fig. 1b, d). These effects, however, were smaller compared to *Smn*+*/*+*;SMN2tgtg* motoneurons, which point to a disturbed actin cytoskeleton in growth cone filopodia of Smn-deficient neurons. Next, we examined whether these differences correlate with differential distribution of F-actin within growth cones in Smn-deficient neurons. We used SIM to analyze the association of axonal ER with F-actin in mCherry-ER expressing motoneurons that were immunostained against F-actin using Phalloidin (Additional file 1: Fig. S1a, b). Line scan analysis showed a strong overlap of ER with F-actin in both the core and filopodia sub-regions of the growth cone in *Smn*+*/*+*;SMN2tgtg* neurons (Additional file 1: Fig. S1c, d). Interestingly, this strong overlap of ER with F-actin was disturbed in the growth cone core and filopodia of *Smn*−/−*;SMN2tgtg* neurons (Additional file 1: Fig. S1c, d). To rule out whether the observed ER and F-actin colocalization was obtained by chance, a defined ROI within the ER channel was rotated 90 degrees and colocalization was assessed by line scan analysis. As shown in Fig. S1c and d, only a partial overlap between ER and F-actin was detectable in both growth cone core and filopodia. To monitor the co-movements of actin and ER in growth cone filopodia, we performed live cell imaging (Additional file 1: Fig. S2a). We used SiR-Actin probes (10 min incubation with 25 nmol) to label F-actin in the filopodia in mCherry-ER expressing neurons. We observed disturbed actin dynamics in filopodia of axonal growth cones of *Smn*−/−*;SMN2tgtg* motoneurons (Additional file 1: Fig. S2b). Similarly, the co-movements of actin and ER in filopodia of Smn-deficient motoneurons were reduced, as shown by multiple kymographs (Additional file 1: Fig. S2c; Additional file 6: Video S5; Additional file 7: Video S6). Then, we performed rescue experiments with lentiviruses overexpressing the human SMN protein (GFP-SMN) or GFP-actin in Smn-deficient neurons. *Smn*+*/*+*;SMN2tgtg* neurons were transduced with a control GFP-expressing lentivirus. Transduced motoneurons were identified by GFP (Fig. 2a), and ER dynamic movements were evaluated by live cell imaging. Interestingly, overexpression of the human SMN rescued ER dynamic movements in growth cone filopodia in *Smn*−/−*;SMN2tgtg* neurons, indicating that loss of Smn function is associated with impaired ER remodeling in axons in SMA (Fig. 2b, c). Similarly, overexpression of actin rescued the impaired ER movements in filopodia in Smn-deficient motoneurons (Fig. 2c).

Taken together, these data indicate that SMN deficiency is associated with the reduced dynamic remodeling of ER within growth cones, which might be due to the impaired dynamics of ER-associated actin filaments in axon terminals.

### Smn-deficient motoneurons show aberrant ribosome cluster formation and delayed local translation in response to BDNF stimulation

**Fig. 3:**
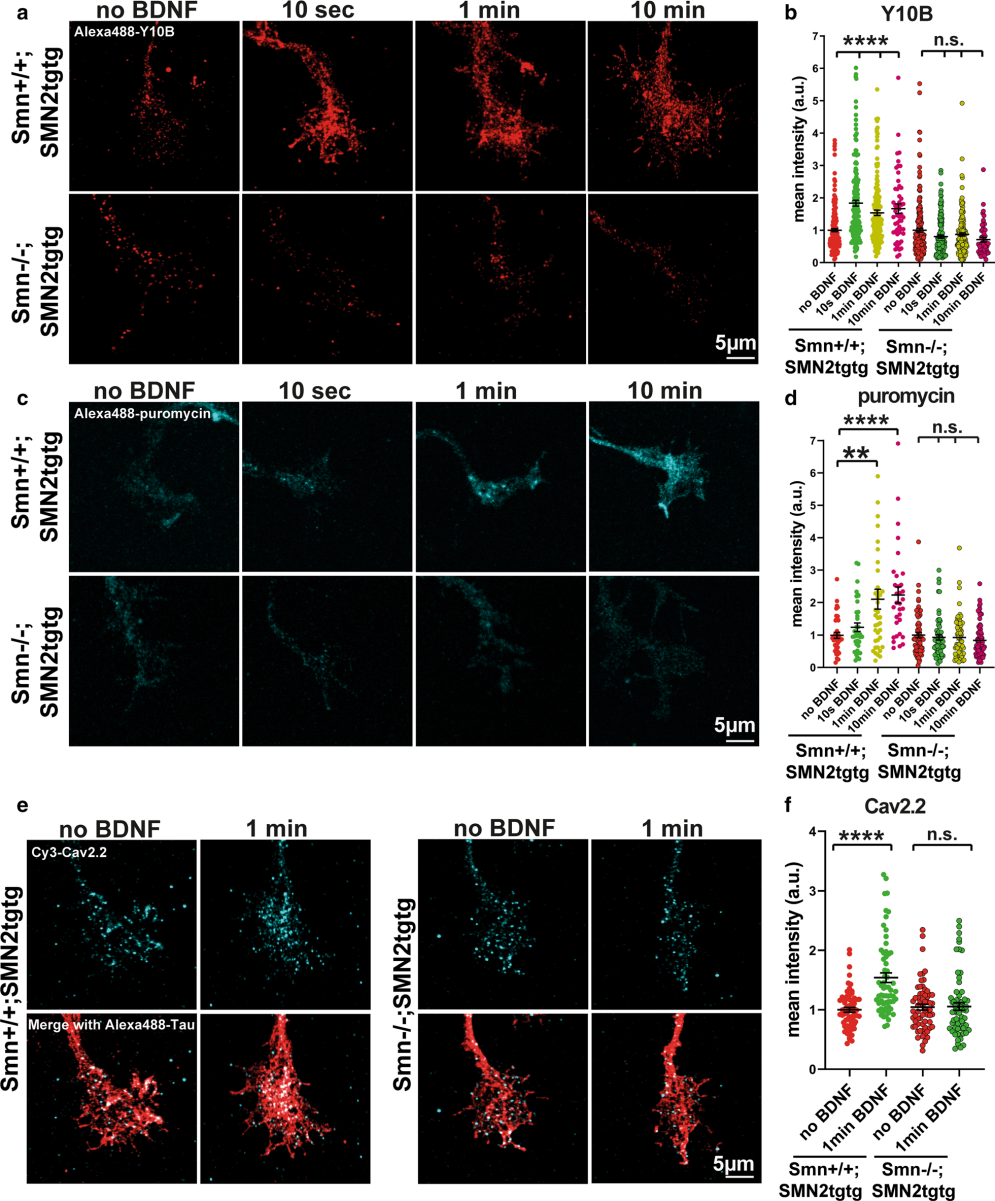
BDNF stimulation does not trigger rapid ribosomal redistribution and protein synthesis in growth cones of Smn-deficient motoneurons. **a** Representative images of growth cones from *Smn*+*/*+*;SMN2tgtg* and *Smn*−/−*;SMN2tgtg* motoneurons immunostained against rRNA using Y10B antibody. **b** Quantification of Y10B mean intensities shows that in contrast to *Smn*+*/*+*;SMN2tgtg* (****P < 0.0001; *n* = 52–214 cells from 3–5 independent experiments), the levels of Y10B do not increase in growth cones of BDNF-stimulated *Smn*−/−*;SMN2tgtg* motoneurons (n.s., *P* = 0.0774; *n* = 51–202 cells from 3–5 independent experiments). **c** Representative images of growth cones of *Smn*+*/*+*;SMN2tgtg* and *Smn*−/−*;SMN2tgtg* motoneurons immunostained against puromycin after BDNF pulse application. **d** Puromycin immunoreactivity does not increase in BDNF-stimulated *Smn*−/−*;SMN2tgtg* (n.s., *P* > 0.9999; *n* = 61–83 cells from 3 independent experiments) compared to *Smn*+*/*+*;SMN2tgtg* neurons (**P = 0.0061; ****P < 0.0001; *n* = 32–39 cells from 2 independent experiments). **e** Representative images of growth cones of *Smn*+*/*+*;SMN2tgtg* and *Smn*−/−*;SMN2tgtg* motoneurons stained against Cav2.2 (cyan) and Tau (red) after 1-min BDNF stimulation. **f** Immunoreactivity of Cav2.2 does not increase in BDNF-stimulated *Smn*−/−*;SMN2tgtg* (n.s., *P* > 0.9999; *n* = 64–66 cells from 3 independent experiments) compared to *Smn*+*/*+*;SMN2tgtg* motoneurons (****P < 0.0001; *n* = 62–64 cells from 3 independent experiments). Data are presented in scatter dot plot; error bars represent mean ± SEM. Statistical analyses were done by One-way ANOVA with Dunn’s post-hoc test

As demonstrated by previous studies, aberrant axonal mRNA localization and impaired local translation are important contributors to disease manifestation and clinical pathology in SMA and ALS [5, 50–52]. Since SMN is involved in the regulation of translation through direct interaction with ribosomes [8], we asked whether the ribosome response to extracellular stimulation is affected upon Smn deficiency. To assess this, we first measured the time course of BDNF-induced ribosome distribution using Y10B immunostaining in growth cones of cultured motoneurons (Fig. 3a). In *Smn*+*/*+*;SMN2tgtg* motoneurons, the area stained for Y10B immunoreactivity increased rapidly within 10 s of stimulation (Fig. 3a, b). This BDNF-dependent cluster formation of Y10B-immunoreactive structures has been recently shown to be caused by actin-dependent redistribution of ribosomes [25]. In contrast, even 10-min stimulation did not elicit any alteration in ribosomal RNA distribution in Smn-deficient growth cones, as the fluorescence intensity and distribution of Y10B did not change (Fig. 3a, b). Next, we stimulated neurons with BDNF and measured the extent of protein synthesis in the growth cone using the puromycin incorporation assay (10-min incubation with 10 µg/ml puromycin) (Fig. 3c). We found that in *Smn*+*/*+*;SMN2tgtg* neurons, BDNF stimulation increased the rate of newly synthesized proteins within a short pulse of 1-min as shown by a significant increase in immunoreactivity of puromycin (Fig. 3c, d). Likewise, we investigated the local synthesis of the transmembrane protein α-1β subunit of N-type Ca^2+^ channels (Cav2.2). We found increased immunoreactivity of Cav2.2 channels in growth cone of *Smn*+*/*+*;SMN2tgtg* neurons following 1-min BDNF stimulation (Fig. 3e, f). We previously showed that this BDNF-induced increase in puromycin and Cav2.2 levels does not rely on the microtubule-dependent axonal transport and could therefore reflect neosynthesis of proteins in the axonal growth cone [25]. However, Smn-deficient motoneurons failed to show any increase in puromycin (Fig. 3c, d) or Cav2.2 levels after BDNF stimulation (Fig. 3e, f). These data suggest that rapid activation and redistribution of ribosomes are not accomplished in Smn-deficient motoneurons, which consequently leads to delayed induction of local translation in response to extracellular stimuli.

### Dynamic assembly of the rough ER is disturbed in the axonal growth cone in SMA

**Fig. 4:**
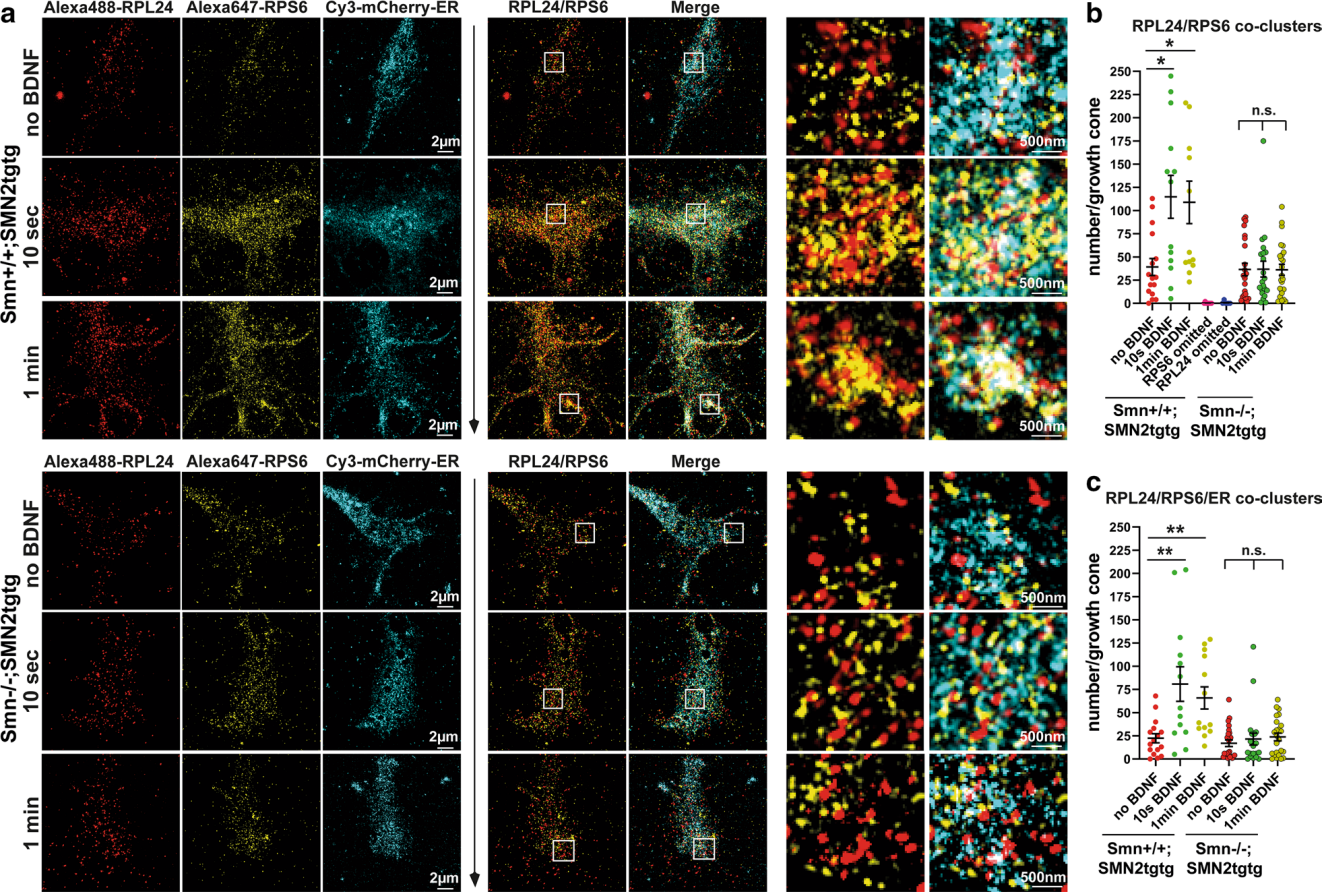
Ribosome responsiveness to the BDNF/TrkB signaling as well as ribosome/ER tethering are impaired in Smn-deficient axon terminals. **a** BDNF-stimulated *Smn*+*/*+*;SMN2tgtg* and *Smn*−/−*;SMN2tgtg* motoneurons expressing mCherry-KDEL were stained against RPL24, RPS6 and mCherry-KDEL. Growth cones were imaged by SIM. White boxes indicate enlarged ROIs within growth cones. In control but not *Smn*−/−*;SMN2tgtg* motoneurons, BDNF induces formation of RPL24/RPS6 co-clusters that colocalize with the axonal ER at 10 s and 1-min poststimulation. **b** *Smn*−/−*;SMN2tgtg* motoneurons do not exhibit increased number of RPL24/RPS6 co-clusters upon stimulation (n.s., *P* = 0.9999; *n* = 21–23 cells), in contrast to *Smn*+*/*+*;SMN2tgtg* (*P = 0.0147 for no BDNF vs. 10-s BDNF; *P = 0.0210 for no BDNF vs. 1-min BDNF; *n* = 13–16 cells from 3 independent experiments). In control experiments, either RPL24 or RPS6 antibodies were omitted to exclude the crosstalk between channels. Colocalization analysis detected almost no co-clusters in the growth cones of control neurons (see also Additional file 1: Fig. S3). **c** In *Smn*+*/*+*;SMN2tgtg* motoneurons, the number of RPL24/RPS6 co-clusters that colocalize with ER increases significantly within 10 s and 1-min of stimulation (**P = 0.0089 for no BDNF vs. 10 s BDNF; **P = 0.0092 for no BDNF vs. 1-min BDNF; *n* = 13–16 cells from 3 independent experiments). In *Smn*−/−*;SMN2tgtg* motoneurons the number of RPL24/RPS6 co-clusters that associate with ER does not increase upon stimulation (n.s., *P* = 0.7601; *n* = 21–24 cells from 3 independent experiments). In all graphs, the average number of co-clusters are shown per growth cone. All representative images are from maximum projection of five 0.12-µm z-stacks. Data are presented in scatter dot plot; error bars represent mean ± SEM. Statistical analyses were done by one-way ANOVA with Dunn’s post-test

**Fig. 5:**
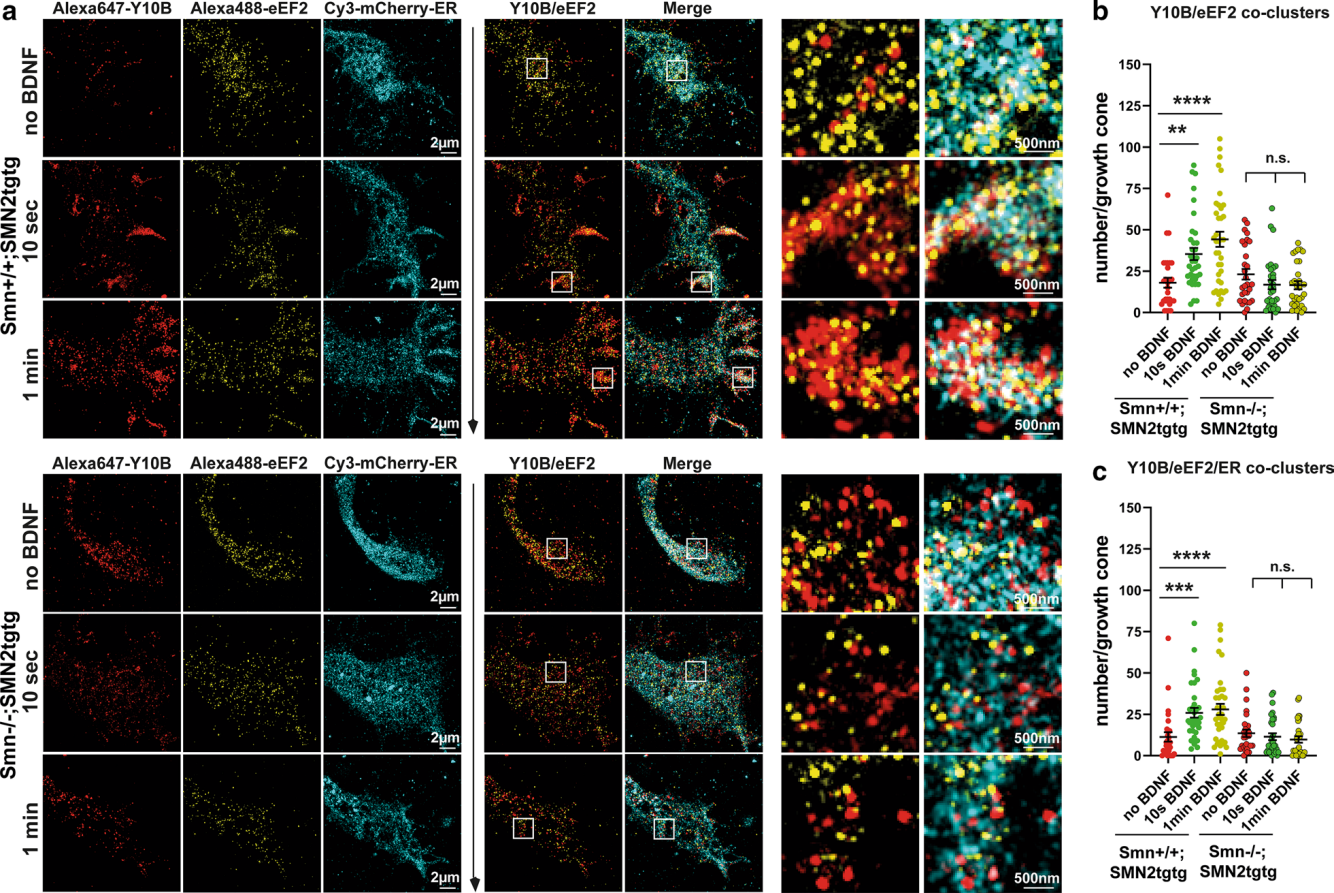
Dynamic assembly of axonal rough ER is defective in Smn-deficient motoneurons. **a** *Smn*+*/*+*;SMN2tgtg* and *Smn*−/−*;SMN2tgtg* motoneurons expressing mCherry-KDEL were immunostained against mCherry-KDEL, Y10B and eEF2. White boxes indicate enlarged ROIs within growth cones. BDNF stimulation induces formation of Y10B/eEF2 co-clusters within 10 s as well as 1-min in control, but not in Smn-deficient motoneurons. **b** Quantification of Y10B/eEF2 co-clusters that are representative of ribosomes in the elongation phase of translation. In contrast to control motoneurons (**P = 0.0023; ****P < 0.0001; *n* = 30–37 cells), *Smn*−/−*;SMN2tgtg* motoneurons do not show an increase in the number of Y10B/eEF2 co-clusters in growth cones (n.s., *P* > 0.9999; *n* = 29–31 cells from 3 independent experiments). **c** Quantification of Y10B/eEF2 co-clusters that colocalize with ER after BDNF pulse application. The number of Y10B/eEF2/ER co-clusters increases within 10 s and 1-min poststimulation in control (***P = 0.0002; ****P < 0.0001; *n* = 28–37 cells), but not in *Smn*−/−*;SMN2tgtg* motoneurons (n.s., *P* = 0.8953; *n* = 23–29 cells from 3 independent experiments). In all graphs, the average number of co-clusters is shown per growth cone. All representative images are from maximum projection of five 0.12-µm z-stacks. Data are presented in scatter dot plot; error bars represent mean ± SEM. Statistical analyses were done by one-way ANOVA with Dunn’s post-hoc test

In the axonal growth cone, rapid responses to extracellular stimuli require fast activation of ribosomes and a dynamic formation of the rough ER in the case of local translation of ion channels and membrane proteins [22, 25]. Our data showing impaired ER movements (Fig. 1) and delayed induction of local translation following BDNF stimulation (Fig. 3c–f) prompted us to examine the alterations in the activation of the translational machinery as well as ribosome/ER association in Smn-deficient motoneurons. To label active translation sites, we immunostained motoneurons expressing mCherry-ER against two ribosomal proteins, RPL24 and RPS6, which label 60S and 40S of the ribosomal subunits, respectively, and imaged the growth cones using SIM (Fig. 4a). The resolution of SIM is ~ 120 nm in the x–y–z [53]. The interaction between RPL24 and RPS6 enables formation of a stable bridge between the two ribosomal subunits [54] and thus could be used as a readout for identifying 80S subunits in monosome or polysome forms, when using super-resolution microscopy approaches such as two-color *d*STORM with a resolution of ~ 20 nm [38, 53]. We evaluated the interaction between RPL24 and RPS6 proteins in growth cones (defined as RPL24/RPS6 co-clusters) using JACoP colocalization analysis and found that in *Smn*+*/*+*;SMN2tgtg* neurons, the number of such RPL24/RPS6 co-clusters increased rapidly within 10 s to 1-min of stimulation at the SIM resolution (Fig. 4a, b). Intriguingly, in Smn-deficient motoneurons, we did not detect elevated levels of RPL24/RPS6 co-clusters under the same conditions upon any time point of stimulation (Fig. 4a, b). To exclude the crosstalk between RPL24 and RPS6 channels, we omitted either RPL24 or RPS6 antibody, which resulted in major decreases in the number of RPL24/RPS6 co-clusters in growth cones (number of co-clusters was close to 0) (Fig. 4b, Additional file 1: Fig. S3a, b). This control experiment is also indicative of the sensitivity of the colocalization analysis method used for these experiments. As previously reported, cycloheximide, when used at low concentrations, traps the ribosomes on mRNAs and keeps the 80S subunits in place during the translation elongation, which should result in detection of more RPL24/RPS6 co-clusters [55]. To examine this possibility, we incubated cells with 10 µg/ml cycloheximide during 1-min BDNF stimulation and also included cycloheximide into the fixation buffer. In the BDNF-free condition, we incubated neurons with the NB medium containing 10 µg/ml cycloheximide for 1-min. We observed a slight increase in the number of co-clusters, particularly in the cycloheximide-treated unstimulated neurons, compared to untreated, unstimulated neurons (Additional file 1: Fig. S3c). This increase was not significant, probably because of the short incubation time of 60 s, in which these ribosomal subunits mostly assemble, and disassembly might not be a major issue yet. Furthermore, we determined the number of RPL24/RPS6 co-clusters that colocalized with the axonal ER by SIM, in order to assess the dynamic assembly of the rough ER (Fig. 4a). The *Smn*+*/*+*;SMN2tgtg* motoneurons showed elevated levels of RPL24/RPS6 co-clusters colocalizing with the ER in the growth cone upon 10-s and 1-min BDNF induction compared to the unstimulated *Smn*+*/*+*;SMN2tgtg* neurons (Fig. 4a, c). In contrast to *Smn*+*/*+*;SMN2tgtg*, we did not observe a significant increase in the number of RPL24/RPS6 co-clusters that were associated with ER in growth cones of the BDNF-stimulated Smn-deficient neurons compared to the unstimulated Smn-deficient neurons (Fig. 4a, c). As demonstrated recently, several ribosomal proteins, in particular RPL24, undergo remodeling and hence are not always fully incorporated into the ribosomes [56–58]. Thus, the immunofluorescence signal detected by the RPL24 antibody might represent the 60S/80S ribosomes, but also the free RPL24 protein. Nevertheless, these studies have reported that the exchange of ribosome composition occurs within a time scale of 1 to 2 h, which is different from the short time scales of 10 to 60 s used here for studying ribosome activation. To confirm whether the newly formed translation sites actively participate in the local translation, we performed additional immunostaining experiments using another ribosomal marker, Y10B, which stains ribosomal rRNA and co-stained against the eEF2 in neurons expressing mCherry-ER (Fig. 5a). Co-clusters of the Y10B and eEF2 were used to identify sites of the translation during the translation elongation phase. We also determined the number of Y10B/eEF2 co-clusters, which colocalized with the ER in growth cones, to evaluate the association of ER with ribosomes in the translation elongation phase. We found that similar to the RPL24/RPS6 co-clusters, the *Smn*+*/*+*;SMN2tgtg* neurons displayed a significant increase in the number of Y10B/eEF2 co-clusters within 10-s as well as 1-min stimulations compared to the unstimulated *Smn*+*/*+*;SMN2tgtg* neurons (Fig. 5a, b). However, the BDNF-stimulated Smn-deficient neurons did not show such rapid increase in the levels of Y10B/eEF2 co-clusters when compared to the unstimulated Smn-deficient neurons (Fig. 5a, b). Likewise, the enhanced levels of Y10B/eEF2 co-clusters associated with ER, which were prominent in *Smn*+*/*+*;SMN2tgtg* neurons, were not detected in the Smn-deficient neurons after a 10-s or 1-min BDNF pulse (Fig. 5a, c).

**Fig. 6:**
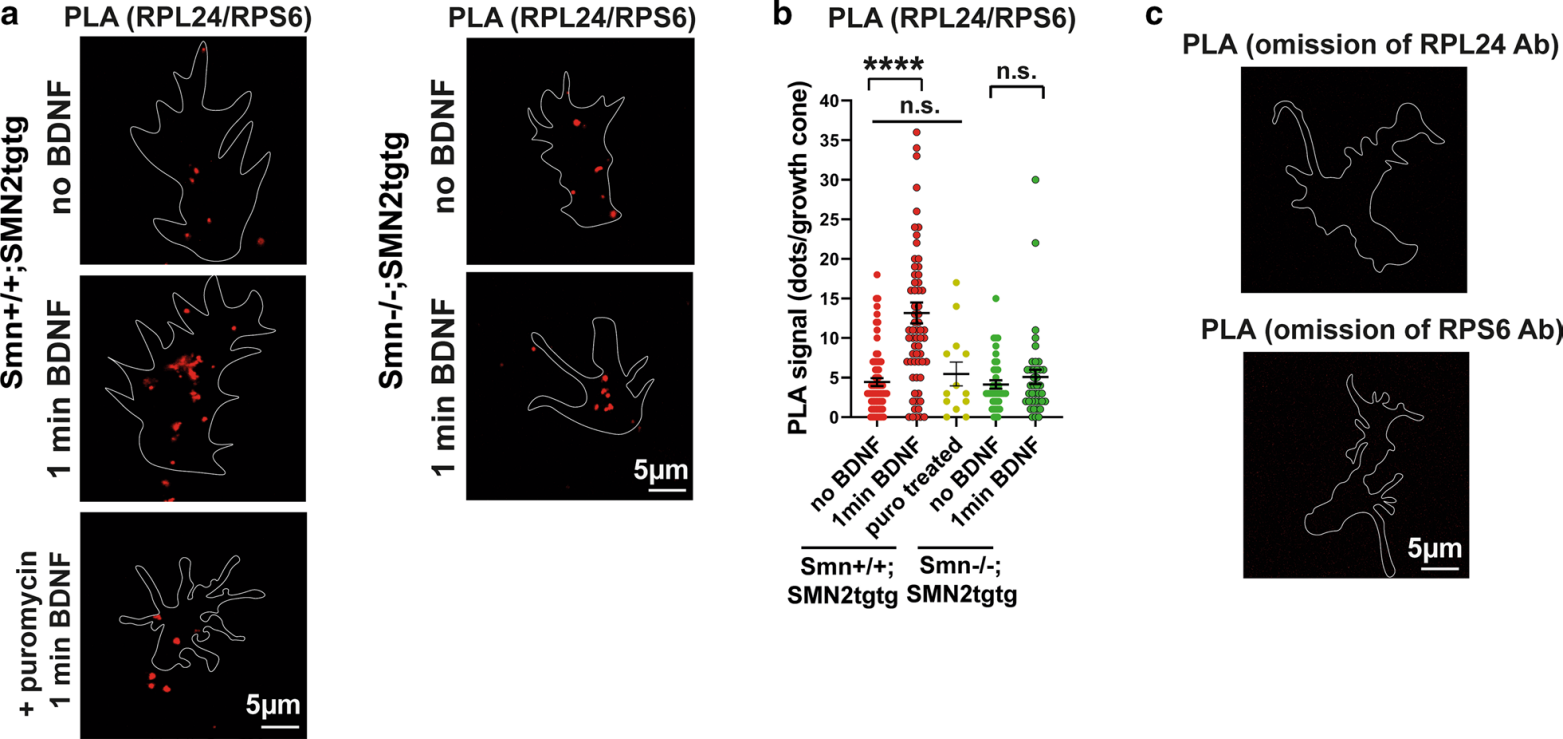
BDNF-induced formation of ribosomal complexes can be visualized by proximity ligation assay. **a** Representative images show PLA signal from RPL24 and RPS6 proteins in growth cones of *Smn*+*/*+*;SMN2tgtg* and *Smn*−/−*;SMN2tgtg* motoneurons following 1-min BDNF stimulation. **b** Increased PLA signal is detectable in growth cones of BDNF-stimulated *Smn*+*/*+*;SMN2tgtg* (****P < 0.0001; *n* = 65–69 cells from 4 independent experiments)*,* but not in *Smn*−/−*;SMN2tgtg* neurons (n.s., *P* = 0.9315; *n* = 39 cells from 3 independent experiments). As a negative control, *Smn*+*/*+*;SMN2tgtg* neurons were pretreated with puromycin before and during the BDNF pulse (**a**). Only a low PLA signal is detectable (n.s., *P* = 0.6882; *n* = 13 cells from 1 experiment). **c** Omitting either the RPL24 or the RPS6 primary antibody  abolishes the PLA signal. Growth cones were outlined using GFP (see the method part for PLA assay). Data are presented in scatter dot plot; error bars represent mean ± SEM. Statistical analyses were done by Two-way ANOVA with Tukey multiple comparison post-hoc test

**Fig. 7:**
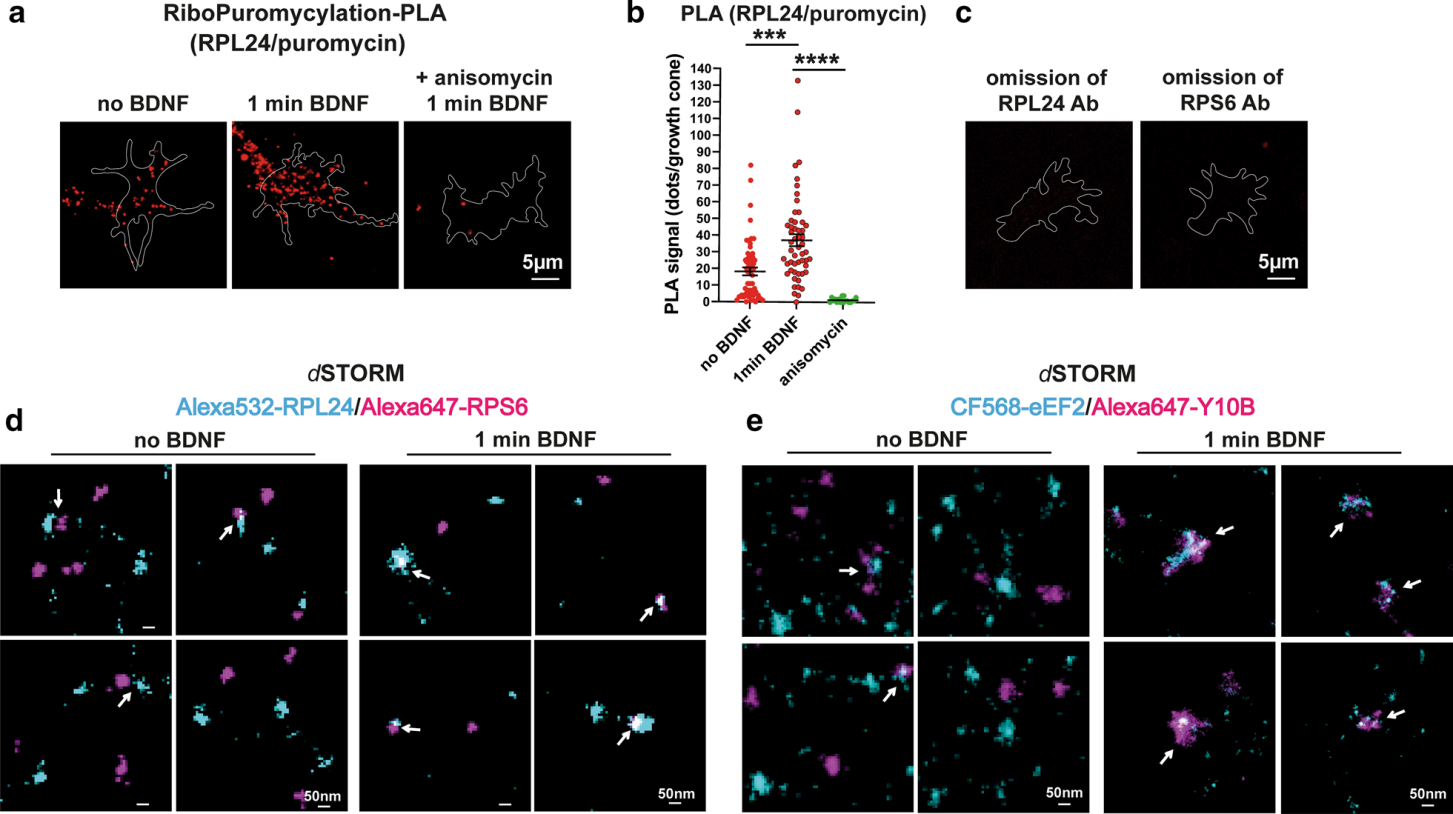
Detection of ribosomal subunit interaction by PLA and two-color *d*STORM. **a** Representative images of RPL24/puromycin-PLA signal detected in growth cones of *Smn*+*/*+*;SMN2tgtg* motoneurons. **b** PLA signal for RPL24/puromycin increases after 1-min BDNF stimulation (***P = 0.0001; *n* = 53–57 cells from 3 independent experiments). In control experiments, anisomycin treatment (**a**) decreased the RPL24/puromycin-PLA signal (****P < 0.0001; *n* = 18 cells from 1 experiment). **c** No PLA signal is detectable in the absence of primary antibodies to RPL24 or puromycin. Growth cones were outlined using GFP (see the method part for PLA assay). **d** and **e** Representative two-color *d*STORM images labeling RPL24 and RPS6 (**d**) and Y10B and eEF2 (**e**) in growth cones of *Smn*+*/*+*;SMN2tgtg* neurons. Upon 1-min BDNF pulse, RPL24 and RPS6 as well as Y10B and eEF2 form clusters that tightly colocalize within growth cones. Arrows indicate positions where the RPL24/RPS6 and eEF2/Y10B signals are located in proximity of 50 nm or less. Data in **b** are presented in scatter dot plot; error bars represent mean ± SEM. Statistical analyses were done by one-way ANOVA with Dunn’s post-hoc test

To validate our SIM data for the RPL24/RPS6 interaction, PLA was performed. In the PLA assay, a signal is detected only when spatial proximity of two interacting proteins is less than 40 nm [12, 34]. Consistent with our SIM data, we detected increased PLA signal following 1-min of BDNF stimulation in the *Smn*+*/*+*;SMN2tgtg* growth cones compared to the unstimulated neurons (Fig. 6a, b). In the S*mn*−/−*;SMN2tgtg* neurons, the amount of PLA signal was not enhanced within 1-min BDNF stimulation (Fig. 6a, b). In control experiments, we pretreated neurons with puromycin (10-min incubation with 200 µg/ml), which inhibits the translation through disassembling 80S subunits [35]. As shown in Fig. 6a, b, upon puromycin treatment, no increase in PLA signal was detected in growth cones of BDNF-stimulated neurons. We also did not detect a PLA signal after omitting either RPL24 or RPS6 antibody (Fig. 6c). To further study the active translation sites in situ, we performed RPM assay with RPL24 and puromycin (Fig. 7a). We first incubated cells with puromycin for 5 min and then performed 1-min BDNF stimulation. In the stimulated *Smn*+*/*+*;SMN2tgtg* neurons, we found an increased PLA signal for RPL24/puromycin, which indicates elevated number of translation sites in growth cones upon extracellular stimulation (Fig. 7a, b). Inhibition of the translation by anisomycin (Fig. 7a, b) or omitting RPL24 or puromycin antibody (Fig. 7c) resulted in detection of low PLA signal, confirming the specificity of the detected RPM-PLA signal. Finally, we performed two-color *d*STORM with a x–y resolution of 20 nm to examine the exact spatial overlap between the two ribosomal subunits 60S and 40S using RPL24 and RPS6 markers. We detected only few overlapping punctae in growth cones of unstimulated neurons, whereas in neurons that received 1-min BDNF pulse, increased numbers of RPL24-RPS6  overlapping punctae were detected (Fig. 7d). These overlapping punctae seemed to be heterogeneous in their dimension, ranging from 50–100 nm. Given that the diameter of the eukaryotic 80S ribosomes is 25 nm [54], these overlapping punctae might represent the 80S subunits in polysome complexes. Similarly, we found increased overlap between Y10B and eEF2 following a short BDNF stimulation (Fig. 7e). These overlapping punctae might indicate ribosomes in the translation elongation phase.

**Fig. 8:**
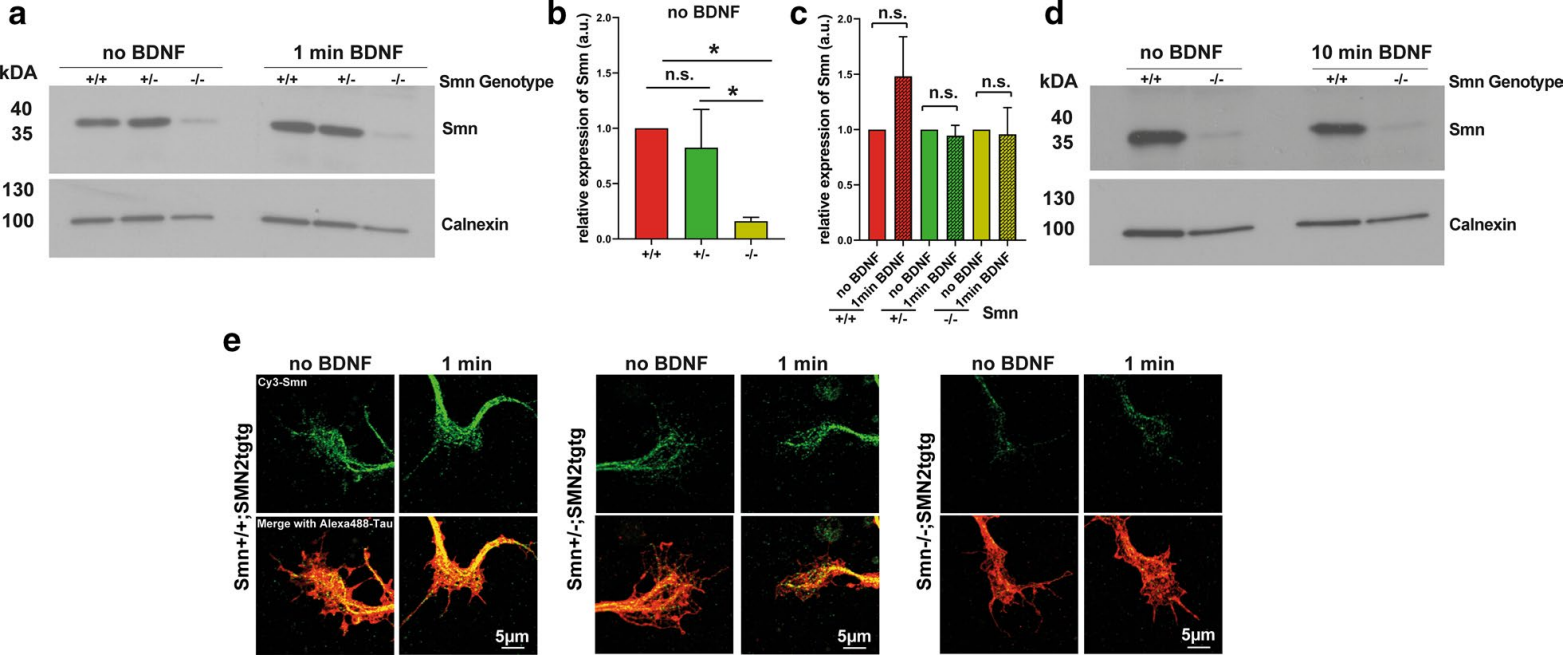
BDNF stimulation does not increase the Smn levels in both control and Smn-deficient motoneurons. **a** Representative Western blots of total protein lysates obtained from cultured *Smn*+*/*+*;SMN2tgtg*, *Smn+/−* *;SMN2tgtg and Smn*−/−*;SMN2tgtg* motoneurons, which have been exposed to 1-min BDNF and probed against Smn and Calnexin. **b** Quantification of Western blot shows reduced total levels of Smn in unstimulated *Smn*−/−*;SMN2tgtg* neurons compared to unstimulated *Smn*+*/*+*;SMN2tgtg* and *Smn+/−*  *;SMN2tgtg* (*P = 0.0500; *n* = 3 independent experiments). **c** Quantification of Western blot shown in **a** indicates no significant increase in Smn levels after 1-min BDNF stimulation in either genotype (n.s., *P* = 0.5000; *n* = 3 independent experiments). **d** Representative Western blot of total protein lysates obtained from *Smn*+*/*+*;SMN2tgtg* and *Smn*−/−*;SMN2tgtg* motoneurons shows no changes in total Smn levels after 10 min BDNF stimulation. **e** Representative images of growth cones of cultured *Smn*+*/*+*;SMN2tgtg*, *Smn+/−* *;SMN2tgtg* and *Smn*−/−*;SMN2tgtg* motoneurons immunostained against Smn (green) and Tau (red) following 1-min BDNF pulse. Smn levels do not increase after 1-min BDNF stimulation in any of the investigated genotypes. Calnexin was used as loading control in **a** and **d,** and for normalization in **b** and **c**. Data are presented in bar diagrams; error bars represent mean ± SEM. Statistical analyses were done by one-tailed Mann Whitney test

As reported recently by Lauria et al., the SMN protein can directly interact with a subpopulation of ribosomes and activate them [8], raising the possibility that the loss of SMN might be responsible for the impaired function of the ribosomes. Notably, no differences were apparent in the number of RPL24/RPS6 and RPL24/RPS6/ER co-clusters (Fig. 4b, c) or in the levels of RPL24/RPS6 PLA signal (Fig. 6b) between unstimulated *Smn*+*/*+*;SMN2tgtg* and unstimulated S*mn*−/−*;SMN2tgtg* neurons. Likewise, the numbers of Y10B/eEF2 as well as Y10B/eEF2/ER co-clusters were comparable in unstimulated neurons from both genotypes (Fig. 5b, c). These observations indicate that in addition to impaired mRNA recruitment onto polysomes upon SMN loss-of-function [8], aberrant responsiveness of the ribosomes to the BDNF/TrkB signaling contributes to disturbed local translation in axon terminals in SMA. Next, we investigated the total Smn expression levels by Western blotting using whole-cell lysates from embryonic cultured *Smn*+*/*+*;SMN2tgtg, Smn*+/-*;SMN2tgtg* and *Smn*−/−*;SMN2tgtg* motoneurons. We could detect only low Smn levels in cell lysates of unstimulated S*mn*−/−*;SMN2tgtg* motoneurons compared to unstimulated *Smn*+*/*+*;SMN2tgtg* and *Smn*+/−*;SMN2tgtg* (Fig. 8a, b). Then we asked whether BDNF stimulation alters the total Smn levels in control and Smn-deficient neurons. Western blotting analysis showed that following 1-min BDNF stimulation, the total Smn levels did not significantly increase in control or in Smn-deficient neurons (Fig. 8a, c). Similarly, 10-min BDNF stimulation did not result in enhanced Smn total protein levels (Fig. 8d). We also examined the levels of Smn protein in axonal growth cones by immunostaining. Consistent with our Western blotting results, we found reduced levels of Smn protein in axonal growth cones of unstimulated S*mn*−/−*;SMN2tgtg* compared to *Smn*+*/*+*;SMN2tgtg* neurons (Fig. 8e), but did not detect increased Smn level upon 1-min BDNF stimulation in growth cones of any of the investigated genotypes (Fig. 8e).

Together, these data suggest that in addition to the altered axonal transcriptome, defective ribosome/ER interplay in the axonal growth cone and consequently delayed initiation of the local translation contribute to diminished stimulus response in axons of motoneurons in SMA.

### Total and surface TrkB levels are not altered in Smn-deficient motoneurons

**Fig. 9:**
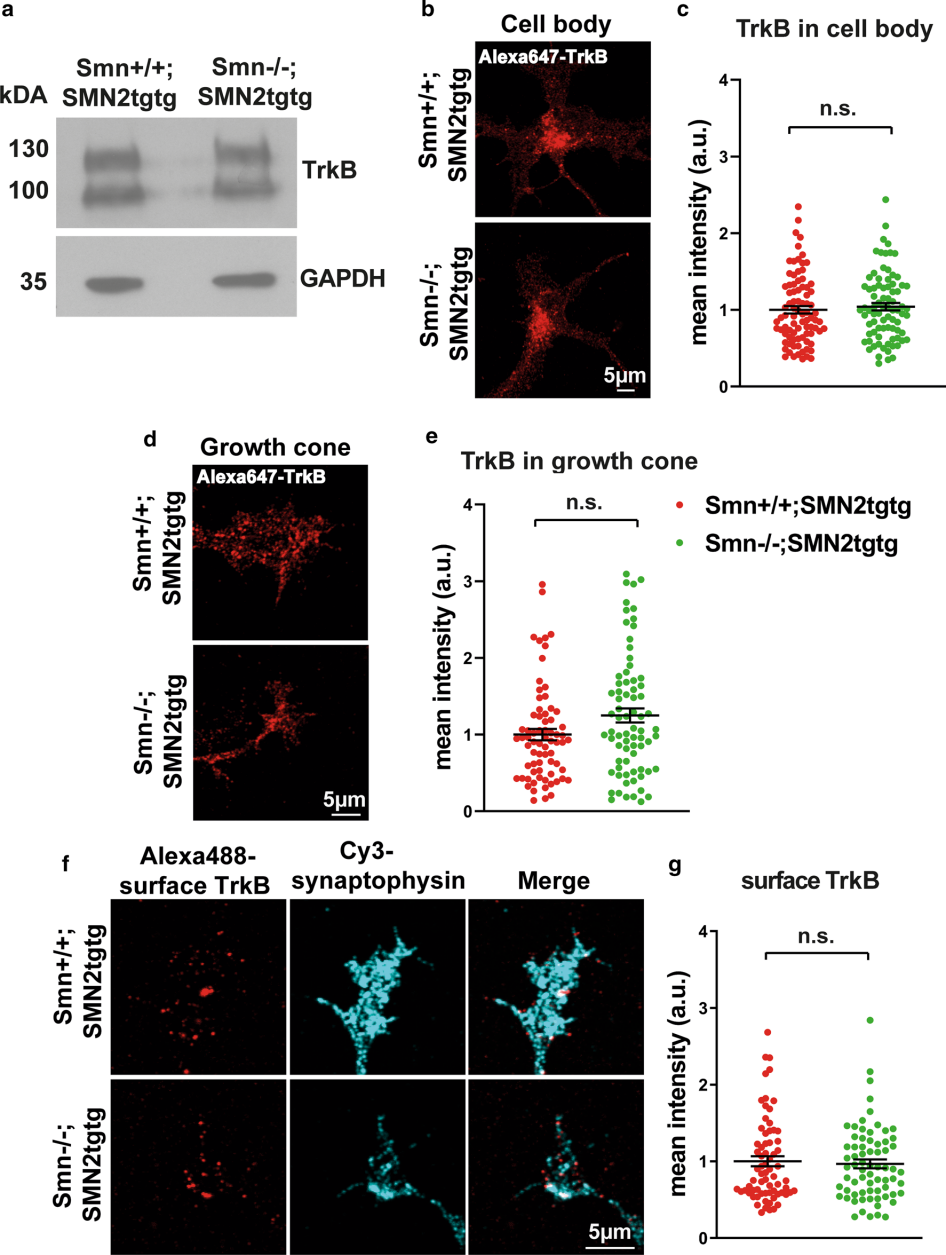
Total and surface TrkB levels are not altered in growth cones of Smn-deficient motoneurons. **a** Representative Western blot of total protein lysates obtained from cultured *Smn*+*/*+*;SMN2tgtg* and *Smn*−/−*;SMN2tgtg* neurons probed against TrkB. GAPDH was used as loading control. **b** Representative images of soma of *Smn*+*/*+*;SMN2tgtg* and *Smn*−/−*;SMN2tgtg* motoneurons that were immunostained against TrkB. **c** Quantification of TrkB signal intensities in the soma shows similar TrkB levels in both *Smn*+*/*+*;SMN2tgtg* and *Smn*−/−*;SMN2tgtg* motoneurons (n.s., *P* = 0.454; *n* = 81–85 cells from 4 independent experiments). **d** Representative images of *Smn*+*/*+*;SMN2tgtg* and *Smn*−/−*;SMN2tgtg* motoneurons showing total TrkB levels in the growth cone. **e** Relatively higher TrkB levels/area in growth cones of *Smn*−/−*;SMN2tgtg* compared to *Smn*+*/*+*;SMN2tgtg* neurons (n.s., *P* = 0.0512; *n* = 71–74 cells from 4 independent experiments). **f** Motoneurons from *Smn*+*/*+*;SMN2tgtg* and *Smn*−/−*;SMN2tgtg* were labeled against surface presented TrkB in the growth cone. Synaptophysin antibody was used to label growth cone boundaries. **g** Graph shows that the surface TrkB levels are comparable in *Smn*+*/*+*;SMN2tgtg* versus *Smn*−/−*;SMN2tgtg* neurons (n.s., *P* = 0.999; *n* = 70 cells from 3 independent experiments). All data are normalized to *Smn*+*/*+*;SMN2tgtg* control. Data are presented in scatter dot plot; error bars represent mean ± SEM. Statistical analyses were done by two-tailed Mann Whitney test

**Fig. 10:**
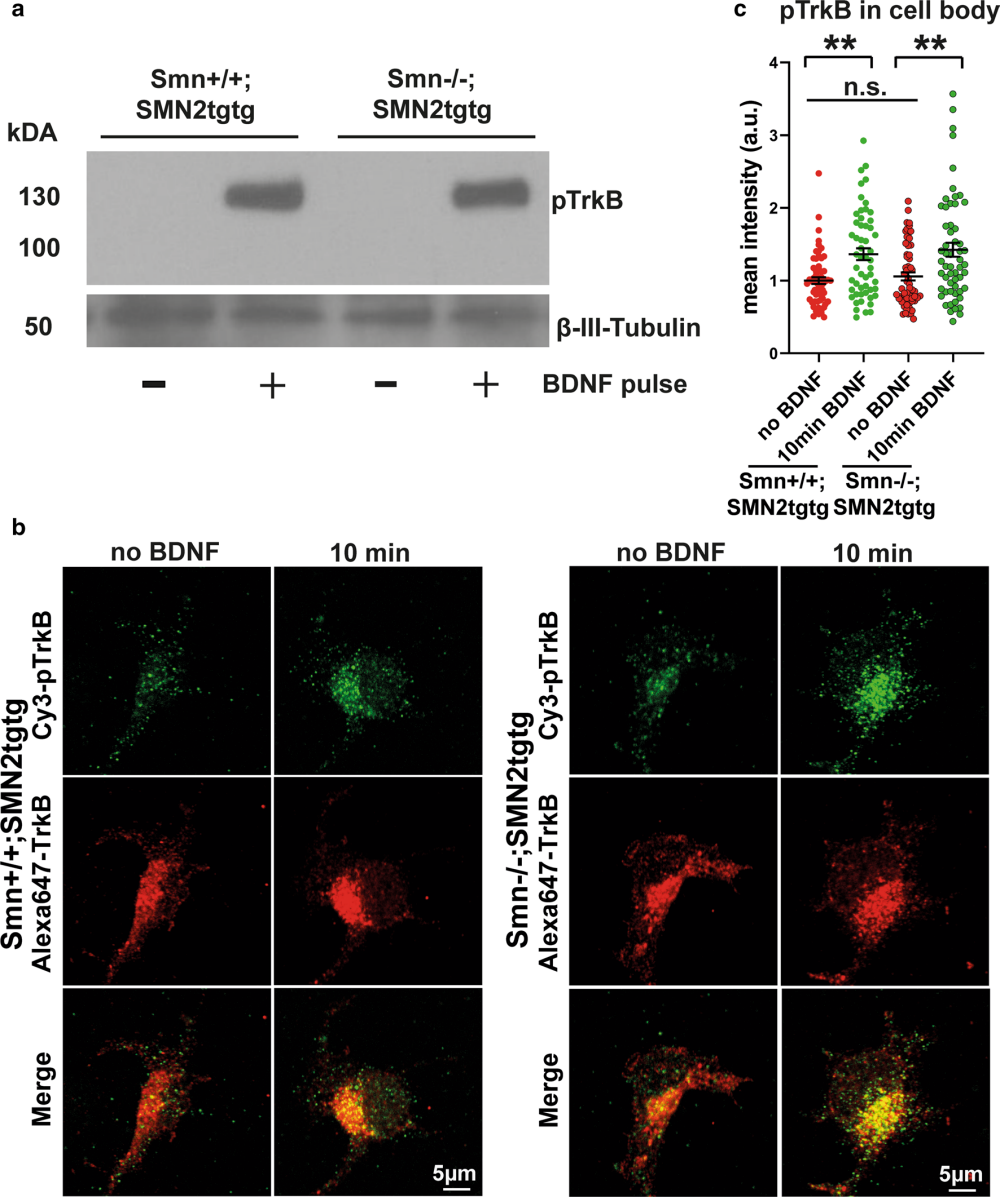
TrkB phosphorylation and activation are intact in Smn-deficient motoneurons. **a** Representative Western blot shows that pTrkB levels increase during 15 min BDNF stimulation in total cell lysates obtained from *Smn*+*/*+*;SMN2tgtg* but also *Smn*−/−*;SMN2tgtg* cultured motoneurons. **b** Representative images show pTrkB levels in the soma of cultured *Smn*+*/*+*;SMN2tgtg* and *Smn*−/−*;SMN2tgtg* motoneurons. **c** Graph shows mean intensities of pTrkB immunoreactivity in the soma. pTrkB levels increase significantly in the soma of *Smn*+*/*+*;SMN2tgtg* (**P = 0.0022; *n* = 54–61 cells) as well as *Smn*−/−*;SMN2tgtg* neurons (**P = 0.0019; *n* = 57–59 cells from 3 independent experiments) after 10 min BDNF pulse. Data are normalized to no BDNF group of *Smn*+*/*+*;SMN2tgtg*. Data are presented in scatter dot plot; error bars represent mean ± SEM. Statistical analyses were done by two-way ANOVA with Tukey multiple comparison post-test

BDNF/TrkB induces the local translation in axon terminals by triggering signaling pathways including MAPK, phosphatidylinositol 3-kinase and the mTOR pathways, thus regulating local protein synthesis and turnover [59–62]. Cultured motoneurons isolated from TrkB knockout mice show similar axonal growth defects as Smn-deficient motoneurons [63]. The defective BDNF-response observed in Smn-deficient neurons suggests either altered TrkB expression or altered TrkB activation or both. To test these possibilities, we first examined TrkB expression in cultured Smn-deficient motoneurons by Western blot analysis and also performed immunostaining of *Smn*+*/*+*;SMN2tgtg* and *Smn*−/−*;SMN2tgtg* motoneurons against TrkB in the soma and growth cone. Strikingly, Western blot data showed that the total TrkB levels were similar in *Smn*+*/*+*;SMN2tgtg* and *Smn*−/−*;SMN2tgtg* neurons (Fig. 9a). Immunostaining assay also showed similar TrkB protein levels in the soma of *Smn*−/−*;SMN2tgtg* neurons compared to control (Fig. 9b, c). The defective response to BDNF might also be due to the altered total TrkB levels in the growth cone or the altered levels of cell-surface TrkB in SMA that ultimately leads to reduced TrkB activation. To test these possibilities, we first evaluated the total TrkB levels in the growth cone by immunostaining (Fig. 9d). Surprisingly, we found slightly increased TrkB levels in growth cones of *Smn*−/−*;SMN2tgtg* neurons that might involve a compensatory upregulation mechanism (Fig. 9e). Next, we carried out a live staining protocol that allows exclusive labeling of TrkB receptors at the cell surface (Fig. 9f). Again, we found similar levels of cell surface TrkB between Smn-deficient neurons and control *Smn*+*/*+*;SMN2tgtg* counterparts, indicating that translocation of TrkB onto cell surface is intact in Smn-deficient motoneurons (Fig. 9g). To examine whether activation of the receptor is altered, we evaluated the phosphorylation of TrkB receptors in Smn-deficient motoneurons by Western blot as well as immunofluorescence assays. As illustrated in Fig. 10a, we detected comparable levels of pTrkB in both Smn-deficient and *Smn*+*/*+*;SMN2tgtg* neurons after BDNF pulse stimulation. In line with that, immunofluorescence experiments using pTrkB antibody confirmed intact TrkB phosphorylation in the soma of Smn-deficient neurons, indicating that TrkB activation in the soma is not affected in SMA (Fig. 10b, c). Hence, the expression and activation of TrkB do not seem to be involved in the perturbed ribosome assembly and dynamic formation of the rough ER in Smn-deficient motoneurons.

## Discussion

The regulation of dynamic remodeling of axonal ER has recently become attentive through studies identifying mutations in ER regulatory proteins that are associated with human neurodegenerative diseases, including hereditary spastic paraplegia, AD, CMT and ALS [26, 64–66]. Here, we examined the dynamic remodeling of ER in the axonal growth cone of isolated motoneurons from a mouse model of SMA and found that the axonal ER movements are severely affected in SMA. Mutations in ER-shaping proteins such as Spastin, ATL1, RTN2 or REEP1 that couple the tubular ER network with microtubule dynamics cause hereditary spastic paraplegia [65, 67]. AD patients carry mutations in Rab10, another protein involved in microtubule and ER coupling [68, 69]. Mutations in vesicle-trafficking protein A and B (VAPA and VAPB) cause late-onset SMA and ALS [64, 70]. VAPA and VAPB proteins reside in the ER, facilitate anchoring of membranous organelles to the ER [64] and regulate tubular ER morphogenesis and dynamics [71]. Similarly, mutations in ER shaping proteins such as FAM134B, BSCL2/seipin, ATL1 and ATL3 cause CMT [18, 72]. Such mutations that impair ER dynamic remodeling do not only disturb ER functions but also impact mitochondria and endosomes through ER membrane contact sites [26]. Our data unravel that loss of Smn protein causes impaired dynamic remodeling of ER in both filopodia and core counterparts of the axonal growth cone in cultured motoneurons. In a previous study, we showed that ER movements, particularly in axonal growth cone filopodia, depend on actin and myosin VI [25]. In line with that, disruption of actin polymerization through CytoD treatment considerably decreased ER dynamics in both growth cone filopodia and core in *Smn*+*/*+*;SMN2tgtg* motoneurons. Intriguingly, in Smn-deficient motoneurons, CytoD treatment had only minor effects on the ER dynamics in growth cone filopodia. These findings point to a disturbed dynamic of F-actin polymerization in axon terminals of Smn-deficient neurons, which is causative of the observed reduced ER movements in this compartment. Moreover, our SIM and live cell imaging data revealed altered colocalization and co-movements of ER and F-actin particularly in the growth cone filopodia of Smn-deficient motoneurons, suggesting disturbed actin-dependent ER entry into filopodia. The actin cytoskeleton appears to play a central role in neurodegeneration through involvement in key axonal functions and synapse maintenance [48]. Indeed, there is emerging evidence for a defective actin cytoskeleton in axons and neuromuscular junctions (NMJs) in SMA patients as well as in animal models [73]. Loss of SMN causes altered axonal transcriptomes and defects in local translation of actin isoforms Actα, Actβ and Actγ in motor axons, resulting in disturbed actin dynamics in the axonal growth cone [74]. Profilin-2 is a neuronal specific regulator of F-actin polymerization, which has been shown to directly interact with the SMN protein [75, 76]. SMN interaction with Profilin-2 reduces its sequestering activity on F-actin, thereby promoting actin polymerization, whereas SMN release or depletion allows Profilin-2 binding to actin monomers, leading to reduced actin polymerization [77]. Moreover, in the absence of SMN, Profilin-2 binds to its upstream kinase ROCK in a competitive manner that in turn leads to its hyperphosphorylation and deactivation [78]. On the other side, reduced binding of ROCK to cofilin and myosin light chain phosphatase results in reduced phosphorylation of cofilin that favors its actin-severing activity [76]. Plastin-3, an actin-bundling protein that also regulates actin turnover, is a protective modifier of SMA and its overexpression has been shown to ameliorate defects in axon growth [79], NMJ maturation and motor functions in SMA animal models [80]. Thus, an impaired actin cytoskeleton might be involved in defective ER dynamics in the growth cone of Smn-deficient motoneurons.

Translational defects and disturbed mRNA recruitment onto polysomes previously reported in SMA mouse models implicate an essential function of the SMN protein in translational regulation [8, 74, 81, 82]. Indeed, SMN is not only involved in the assembly of snRNP particles, but also associates directly with actively translating polysomes, as shown in cultured cells, spinal cord and brain tissues [8, 83]. Our data in particular with two-color *d*STORM and RiboPuromycylation-PLA suggest that, in axon terminals, ribosomal subunits undergo rapid assembly to form functional translation sites in response to a short BDNF stimulation. These ribosomal complexes seem to rapidly translocate to the axonal ER within 10 s after BDNF stimulation. This fast response of ribosomes and ER assures a rapid modulation of the axonal proteome, as the locally translated transmembrane proteins such as Cav2.2 become visible within 1-min poststimulation. The Smn-deficient motoneurons fail to respond to BDNF stimuli by eliciting the formation of ribosomal complexes, which ultimately results in delayed translation initiation. This occurs despite the presence of TrkB at sufficient levels and the activation of TrkB through BDNF on the cell surface. In addition, in Smn-deficient neurons, the rapid translocation of ribosomes toward the axonal ER does not accomplish and the rough ER does not form after BDNF pulse stimulation. Interestingly, formation of ribosomal complexes and their ER tethering depend on the actin cytoskeleton as treatment with cytoD impedes the rapid formation of new translation sites and their translocation onto axonal ER in response to the BDNF pulse [25]. In line with previous studies reporting that actin mRNAs translate on the SMN-primed ribosomes [8] and that local translation of actin isoforms is disturbed upon Smn loss [6, 74], we propose that the perturbed actin dynamics is responsible for the defective ribosome cluster formation and assembly of rough ER in axons in SMA. Our live cell imaging data showing that the effect of Smn deficiency on ER dynamic remodeling in filopodia could be rescued by either SMN or actin overexpression convincingly add evidence to this hypothesis.

In motoneurons, BDNF modulates diverse neuronal functions such as survival, axon growth and differentiation [84–86]. Phosphorylation and activation of the TrkB receptor is crucial for the survival of motoneurons [87]. Strikingly, we did not detect alterations in the TrkB total protein level in both growth cone and soma of Smn-deficient motoneurons. Similarly, live staining of the surface TrkB showed comparable levels of the TrkB receptor in the plasma membrane of Smn-deficient *versus* control motoneurons, indicating that TrkB expression and surface translocation are not disturbed in SMA. Finally, stimulation with BDNF could trigger the phosphorylation of the TrkB in Smn-deficient motoneurons. Importantly, we did not detect any significant increase in the total Smn level or its axonal localization, even in control neurons after 10-min BDNF stimulation. Thus, an impaired actin cytoskeleton affecting ER dynamic remodeling and ribosome/ER anchoring appears to underlie the disturbed BDNF responsivity and synaptic defects in SMA.

## Conclusions

In summary, our study on Smn-deficient motoneurons identified altered dynamics of the ribosome–ER interplay as well as disturbed ER dynamic remodeling in axon terminals. This appears to correlate with altered actin distribution and altered responses of actin dynamics to extracellular stimuli. This observation could explain why lack of SMN causes defects in synaptic differentiation at postnatal stages when signals from muscle, Schwann cells and extracellular matrix contribute to full differentiation of functional neuromuscular junctions.

## Supplementary Information

**Additional file 1: Fig. S1.** ER and F-actin colocalization is disturbed in growth cones of Smn-deficient motoneurons.** Fig. S2.** Co-movements of ER and F-actin are reduced in growth cone filopodia of Smn-deficient motoneurons. **Fig. S3.** No crosstalk is detectable between RPL24 and RPS6 channels.

**Additional file 2: Video S1.** ER movements in filopodia of the axonal growth cone of *Smn−/−;SMN2tgtg* motoneurons. *Smn−/−;SMN2tgtg* motoneurons expressing mCherry-ER were imaged for 15 min at 2 sec intervals to visualize ER in the growth cone using an epifluorescence microscope. Related to Fig. 1.

**Additional file 3: Video S2.** ER movements in filopodia of the axonal growth cone of *Smn+/+;SMN2tgtg* motoneurons. *Smn+/+;SMN2tgtg* motoneurons expressing mCherry-ER were imaged for 15 min at 2 sec intervals to visualize ER in the growth cone using an epifluorescence microscope. Related to Fig. 1.

**Additional file 4: Video S3.** ER movements in axonal growth cone core of *Smn−/−;SMN2tgtg* motoneurons.* Smn−/−;SMN2tgtg* motoneurons expressing mCherry-ER were imaged for 15 min at 2 sec intervals to visualize ER in the growth cone using an epifluorescence microscope. Related to Fig. 1.

**Additional file 5: Video S4.** ER movements in axonal growth cone core of *Smn+/+;SMN2tgtg* motoneurons. *Smn+/+;SMN2tgtg* motoneurons expressing mCherry-ER were imaged for 15 min at 2 sec intervals to visualize ER in the growth cone using an epifluorescence microscope. Related to Fig. 1.

**Additional file 6: Video S5.** ER and actin co-movements in axonal growth cone filopodia of *Smn+/+;SMN2tgtg* motoneurons.* Smn+/+;SMN2tgtg* motoneurons expressing mCherry-ER were stained with SiR-Actin to visualize actin and ER co-movements in growth cone filopodia. Images are taken for 5 min at 2 sec intervals using an epifluorescence microscope. Related to Fig. S2.

**Additional file 7: Video S6.** ER and actin co-movements in axonal growth cone filopodia of *Smn−/−;SMN2tgtg* motoneurons. *Smn−/−;SMN2tgtg* motoneurons expressing mCherry-ER were stained with SiR-Actin to visualize actin and ER co-movements in growth cone filopodia. Images are taken for 5 min at 2 sec intervals using an epifluorescence microscope. Related to Fig. S2.

## Data Availability

The primary datasets used and/or analysed during the current study are available from the corresponding author on reasonable request.

## Abbreviations

AD: Alzheimer’s disease
ALS: Amyotrophic lateral sclerosis
BDNF: Brain-derived neurotrophic factor
CMT: Charcot–Marie–Tooth disease
ER: Endoplasmic reticulum
HSP: Hereditary spastic paraplegia
NMJs: Neuromuscular junctions
SIM: Structured illumination microscopy
SMA: Spinal muscular atrophy
SMN: Survival of motor neuron
TrkB: Tropomyosin receptor kinase B
