# Supplementary material for: Impaired dynamic interaction of axonal endoplasmic reticulum and ribosomes contributes to defective stimulus–response in spinal muscular atrophy
Source: Transl Neurodegener. 2022 Jun 2;11:31. doi: 10.1186/s40035-022-00304-2 (PMC9161492; doi:10.1186/s40035-022-00304-2)
Supplement: Supplementary file 1 — Additional file 1: Fig. S1. ER and F-actin colocalization is disturbed in growth cones of Smn-deficient motoneurons. Fig. S2. Co-movements of ER and F-actin are reduced in growth cone filopodia of Smn-deficient motoneurons. Fig. S3. No crosstalk is detectable between RPL24 and RPS6 channels. [file 40035_2022_304_MOESM1_ESM.docx]

**Additional file 1**


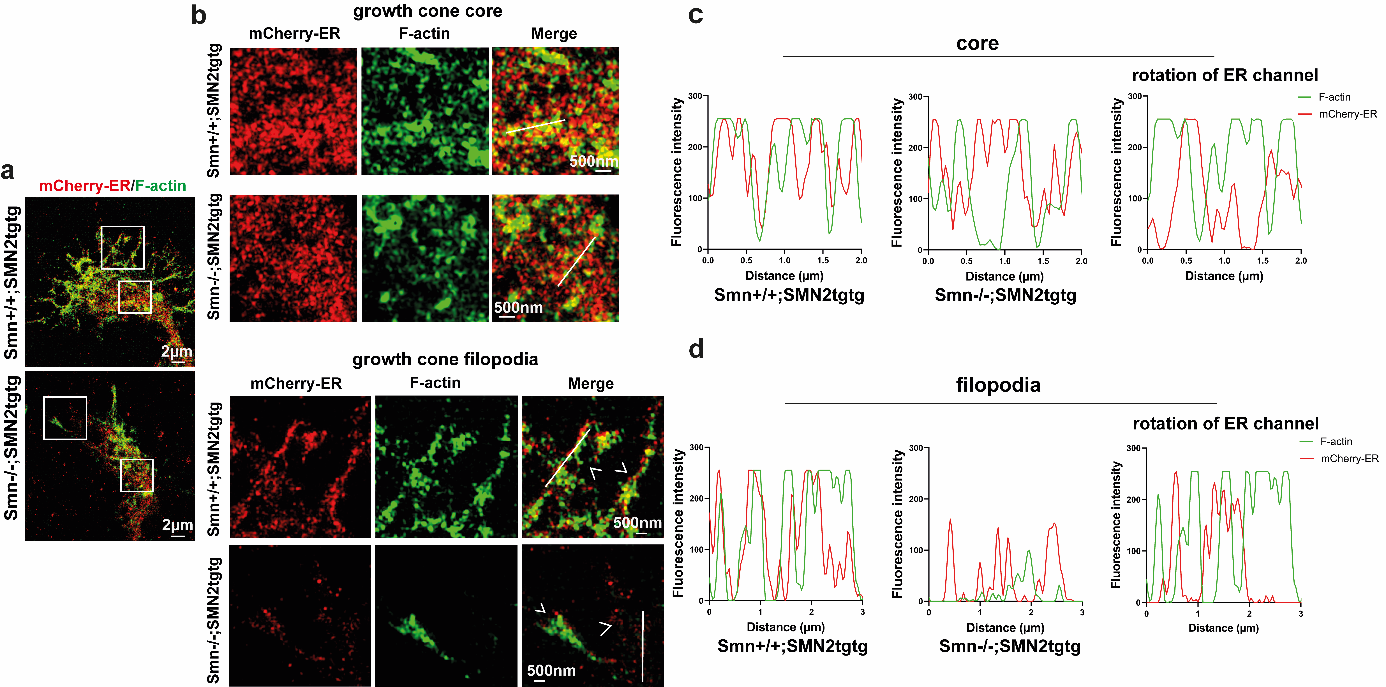
 Supplementary Figure 1.

**ER and F-actin colocalization is disturbed in growth cones of Smn-deficient motoneurons.** **a** and **b** *Smn+/+;SMN2tgtg* and *Smn-/-;SMN2tgtg* motoneurons were transduced with lentiviruses expressing mCherry-KDEL (mCherry-ER) to visualize ER and immunostained against mCherry and F-actin using Phalloidin and imaged by SIM. **a** Overview of representative SIM images of mCherry-ER and F-actin in growth cones of *Smn+/+;SMN2tgtg* versus *Smn-/-;SMN2tgtg* motoneurons. White squares indicate ROIs within growth cone core or filopodia. **b** Images show enlarged ROIs from **a**. ER is labeled in red and F-actin is labeled in green. **c** Line scan diagrams were used to quantify the colocalization of ER with F-actin within growth cone cores of *Smn+/+;SMN2tgtg and Smn-/-;SMN2tgtg* neurons. Line scan diagrams in **c** correspond to the drawn line in **b**. As control, ER channel was rotated 90 degrees, which resulted in only a partial overlap of ER and F-actin in the core (the third diagram). **d** Line scan diagrams show colocalization of the ER with F-actin in the growth cone filopodia of *Smn-/-;SMN2tgtg* neurons compared to the control. Rotation of the ER channel in filopodia revealed only a partial colocalization of ER with F-actin (the third diagram). Representative images are from maximum projection of five 0.12 µm z-stacks.


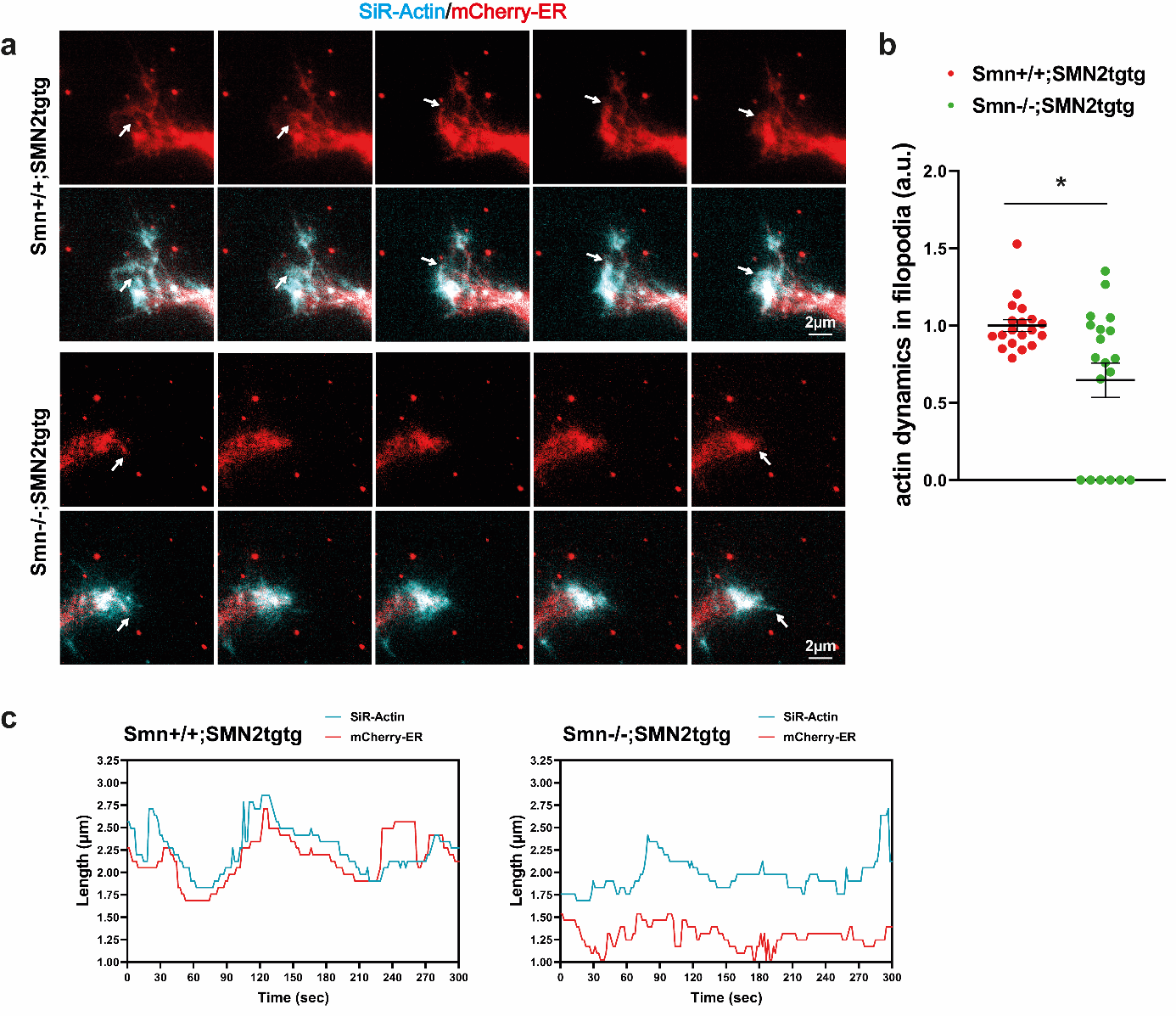


Supplementary Figure 2.

**Co-movements of ER and F-actin are reduced in growth cone filopodia of Smn-deficient motoneurons. a** Representative time lapse images of growth cone filopodia of *Smn+/+;SMN2tgtg* and *Smn*-/-*;SMN2tgtg* motoneurons expressing mCherry-ER that are co-labeled with SiR-Actin. **b** Quantification by Image Correlation Spectroscopy shows reduced actin dynamics in growth cone filopodia of *Smn*-/-*;SMN2tgtg* neurons (***,** P = 0.0226; n = 19 growth cones from 2 independent experiments). Shown are average actin dynamics in filopodia per growth cone. **c** Disturbed coordinated ER and actin co-movements in growth cone filopodia of *Smn*-/-*;SMN2tgtg* neurons. Arrows indicate sites of active movements of SiR-Actin and nCherry-ER. All data are normalized to *Smn+/+;SMN2tgtg* control. Data are presented in scatter dot plot; error bars represent mean ± SEM. Statistical analyses were done by two-tailed Mann Whitney test.


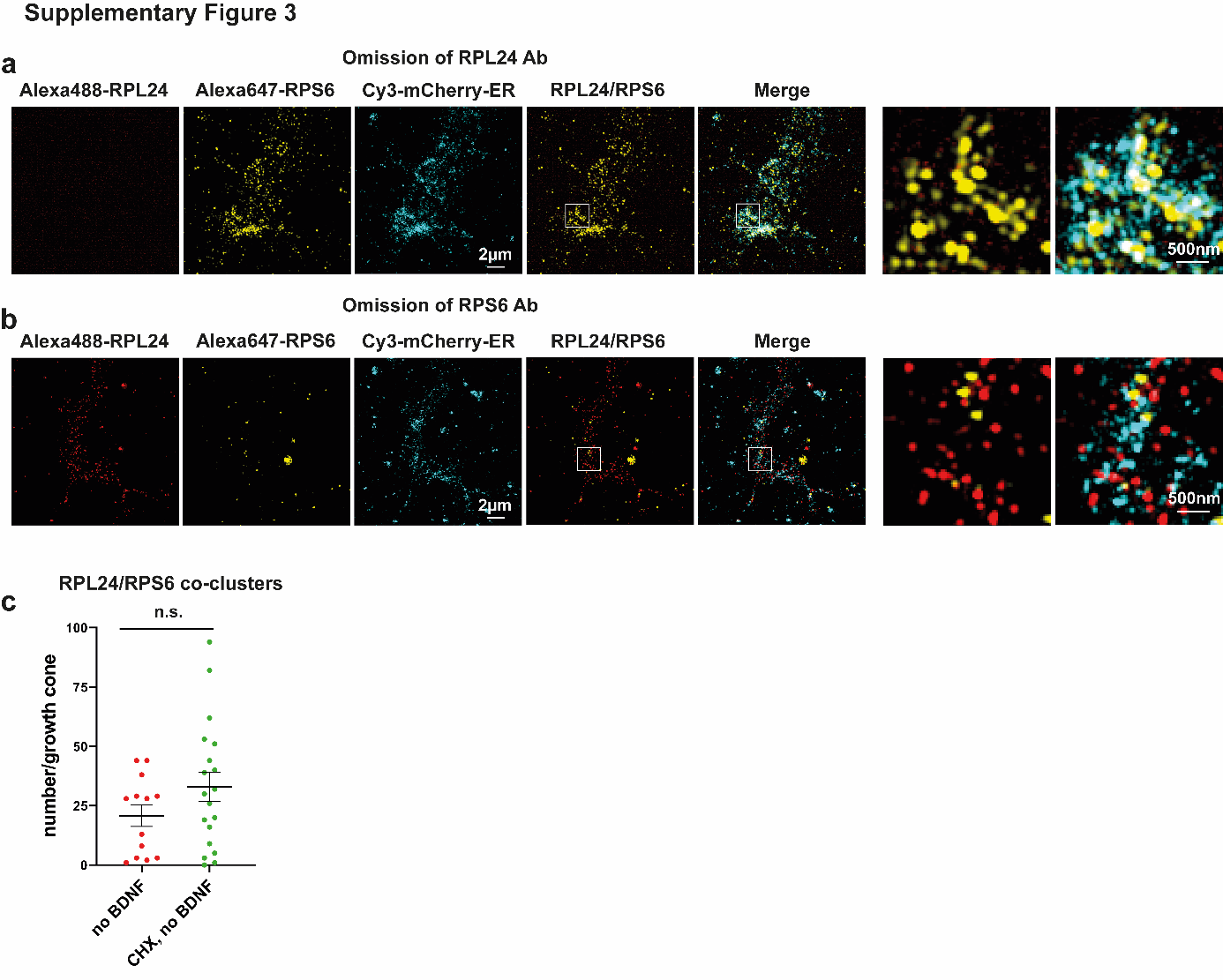


Supplementary Figure 3.

**No crosstalk is detectable between RPL24 and RPS6 channels. a** and **b** To exclude a possible crosstalk between RPL24 and RPS6 channels, primary antibodies to RPL24 (**a**) or RPS6 (**b**) were omitted and motoneurons were incubated only with the secondary antibodies. No colocalization was observed between RPL24 and RPS6 secondary antibodies in growth cones from either condition. Motoneurons expressed mCherry-ER to visualize growth cones. **c** 1 min cycloheximide treatment slightly but not significantly increases the number of RPL24/RPS6 co-clusters in growth cones (n.s., P = 0.2263 ; n = 13-19 cells from 2 independent experiments). Data in **C** are presented in scatter dot plot; error bars represent mean ± SEM. Statistical analyses were done by two-tailed Mann Whitney test.
